# Recent Advances in Cerium Oxide-Based Memristors for Neuromorphic Computing

**DOI:** 10.3390/nano13172443

**Published:** 2023-08-28

**Authors:** Sarfraz Ali, Muhammad Abaid Ullah, Ali Raza, Muhammad Waqas Iqbal, Muhammad Farooq Khan, Maria Rasheed, Muhammad Ismail, Sungjun Kim

**Affiliations:** 1 Department of Physics, Riphah International University, Lahore Campus, 13-KM Raiwand Road, Lahore 54000, Pakistan; 2 Department of Physics, University of Okara, Okara 56300, Pakistan; 3 Department of Physics “Ettore Pancini”, University of Naples ‘Federico II’, Piazzale Tecchio, 80, 80125 Naples, Italy; 4 Department of Electrical Engineering, Sejong University, Seoul 05006, Republic of Korea; 5 Department of Advanced Battery Convergence Engineering, Dongguk University, Seoul 04620, Republic of Korea; 6 Division of Electronics and Electrical Engineering, Dongguk University, Seoul 04620, Republic of Korea; ismailmalikbzu10@gmail.com

**Keywords:** CeO_2_, RRAM memristive devices, filamentary mechanisms, symmetric and asymmetric electrodes, switching layers, capping layers, neuromorphic learning systems, synaptic devices

## Abstract

This review article attempts to provide a comprehensive review of the recent progress in cerium oxide (CeO_2_)-based resistive random-access memories (RRAMs). CeO_2_ is considered the most promising candidate because of its multiple oxidation states (Ce^3+^ and Ce^4+^), remarkable resistive-switching (RS) uniformity in DC mode, gradual resistance transition, cycling endurance, long data-retention period, and utilization of the RS mechanism as a dielectric layer, thereby exhibiting potential for neuromorphic computing. In this context, a detailed study of the filamentary mechanisms and their types is required. Accordingly, extensive studies on unipolar, bipolar, and threshold memristive behaviors are reviewed in this work. Furthermore, electrode-based (both symmetric and asymmetric) engineering is focused for the memristor’s structures such as single-layer, bilayer (as an oxygen barrier layer), and doped switching-layer-based memristors have been proved to be unique CeO_2_-based synaptic devices. Hence, neuromorphic applications comprising spike-based learning processes, potentiation and depression characteristics, potentiation motion and synaptic weight decay process, short-term plasticity, and long-term plasticity are intensively studied. More recently, because learning based on Pavlov’s dog experiment has been adopted as an advanced synoptic study, it is one of the primary topics of this review. Finally, CeO_2_-based memristors are considered promising compared to previously reported memristors for advanced synaptic study in the future, particularly by utilizing high-dielectric-constant oxide memristors.

## 1. Introduction

Because of the variety of oxide materials, the resistive-switching (RS) mechanism is widely investigated in exploring resistance variation processes in memristors. However, improving RS mechanisms attributed to resistive random-access memories (RRAMs) is still elusive. Nevertheless, no crucial switching mechanism has been guaranteed to be ideal, i.e., 100% RS behavior, to date. Therefore, an extensive review is indispensable for elucidating the switching kinetics of the desired switching materials to enable the incorporation of synaptic devices into these studies, which is presented in this work [1,2,3]. More recently, memristive devices have displayed new opportunities in neuromorphic computing research [4,5,6]. RS devices’ mechanism has been significantly explored in various nanomaterials such as TiO_2_, Cr_2_O_3_, and FeO_x_ [7,8,9], and in high-k dielectrics with large bandgaps such as Al_2_O_3_ and HfO_2_ [10]. As an illustration of pursuing a comprehensive understanding of neuromorphic dynamics, an intricately developed computational model was introduced, specifically focusing on Ag-PVP nanowire networks (NWNs). In this context, Ag/PVP/Ag junctions were established at the interfacial contacts between the nanowires. Notably, all electrical junctions were meticulously modeled as voltage-controlled memristors. Within this framework, investigation into the switching mechanisms encompassed various aspects, outlined under headings such as “Collective Junction Switching Drives Non-local Transport”, “Avalanche Switching Dynamics”, and “Order-Chaos Transition from Polarity-Driven Switching” [11]. However, they lacked an approach to achieve the desired RS behavior in memory technology. For example, electrode engineering, unipolar, bipolar (symmetric and asymmetric), and threshold behaviors are still questionable. Moreover, they do not offer remarkable outcomes in their filamentary mechanism in insulating layers containing metal/insulator/metal and metal/insulator/insulator/metal configurations, as reported in the literature. Meanwhile, among oxide-based materials, cerium oxide (CeO_2-x_)-based nanomaterials possess potential applications such as catalysts, solar cells, and gate nanomaterials belonging to metal oxide-based semiconductor devices [12]. Nevertheless, they still show limitations in achieving the targeted outcomes. There was also a focus on single-crystalline materials owing to the allowed disentangling defect behavior occurring at grain boundaries, which are considered the intrinsic property of bulk materials [13].

To date, cerium oxide (CeO_2_) has been suggested as a promising candidate showing analog-switching behavior of memristors. Furthermore, several researchers have reported significant digital-type resistance variation for RRAM implementation using a ceria switching layer [14,15,16,17]. Ismail et al. [16] demonstrated the bipolar switching mechanism in Al/CeO_2_/Au and Zr/CeO_x_/Pt architectures. Lin et al. also presented the nonpolar switching behavior due to the polycrystalline CeO_2_ layer of a Pt/CeO_2_/Pt structure [17]. Remarkably, CeO_2_-based analog switching is still being researched. In this respect, the demonstration of analog-based switching of a Pt/CeO_2_/Pt memristor has been displayed in memristive technology. Moreover, Pt/CeO_2_/Pt analog synaptic devices also possess a symmetric electrode structure, thereby prominently exhibiting emulated synaptic trends in the form of potentiation and depression behaviors in computation. Therefore, its comprehensive switching peculiarities for artificial synapses have been extensively studied [18]. In previous studies, an identical synaptic weight modulation has been prominently considered owing to the functioning of the neural setup for generating voltage pulses containing symmetric amplitude as well as bipolar functioning toward potentiation and depression trends. Again, analog resistance variations exhibit long-term stability (LTS) to ensure constancy in signal formation, simulated learning, and retention processes. The abovementioned properties have been achieved under low-power/energy consumption conditions, as they always remain the primary goal of research areas [19].

In addition, a Pt/ITO/CeO_2_/Pt memristive device and ITO behaving as a capping layer often play a vital role toward synaptic trends. The stability of analog resistance variation to emulate the potentiation and depression functions in an ITO free-layered Pt/CeO_2_/Pt memristor has been elaborately reported [18,20]. Further, the exhibited potentiation and depression dynamics of the Pt/CeO_2_/Pt memristor have shown nonlinearity and asymmetry instead of a symmetric architecture. Because it has a nonfilamentary route such as that of other memristors, this device also has the disadvantage of memory loss, which limits its stability in analog/multilevel synaptic weight modulation. However, a CeO_2_-based memristor having oxygen reactivity in an ITO electrode form exhibits enhanced conductivity caused by increased density of oxygen vacancies (O_V_s) [21]. Hence, oxygen ions (O^2−^) are exchanged where CeO_2_-ITO interfacial contact occurs in the Pt/ITO/CeO_2_/Pt device, leading to stability of resistance modulation. Therefore, improved linear behavior, symmetric potentiation and depression, and long-life stability are the fundamental steps in promoting the Pt/ITO/CeO_2_/Pt memristor for artificial synapse devices in neuromorphic systems [19].

Further, memristors are suggested as candidates for artificial synapses owing to comparable structures with presynaptic-neuron/synapse/postsynaptic-neuron architectures with higher density of neuromorphic synaptic systems. Furthermore, their resistance states always mimic analog synaptic weight functioning with a wide variety of synaptic dynamics, such as postsynaptic current, paired-pulse facilitation (PPF), short-term plasticity (STP), long-term plasticity (LTP), and spike-time-dependent plasticity (STDP) [22,23,24]. Herein, a promising analog synapse formation in the Pt/CeO_2_/Pt memristor under annealing of ceria layer has also been investigated to explore optimal effect of Pt/CeO_2_ interfacial states. In that study, the primary resistance change was attributed to the tuning of interfacial states to mimic analog synaptic weight modulation in biological synapses without involving filament formation [24]. Additionally, the CeO_2_ layer complemented the Si-based electronics in the synapse layer, showing a reasonable permittivity of ~26 with a bandgap of ~3.2 eV [25] and with fruitful outcomes for the integrated artificial synapses decorated over a Si-based neural hardware foundation.

For the advancement in analog resistance variation in Pt/CeO_2_/Pt memristors, an approach involving altering the post-deposition annealing environment for changing the switching parameters of the CeO_2_ synapse-based layer has been widely studied. However, from the conduction mode investigation data and spectroscopic study of chemical-bonding states, the reference device was found to be capable of voltage-controlled operation due to Pt/CeO_2_ interfacial contacts [26]. Furthermore, researchers have worked on many artificial intelligence-based devices showing functions identical to those of neurons to function in several tasks such as visual recognition, audio recognition, and artificial nociceptor functioning [27,28,29].

Memristors have been extensively discussed as capable artificial synaptic devices owing to inheriting synaptic weight change into neural networks [13,18]. Moreover, synaptic plasticity can also be modulated by various input signals as synaptic functions. In this regard, various oxide-based nanomaterials, such as ZnO [30], Y_2_O_3_ [31], CeO_2_ [32,33,34], HfO_x_ [35], and ZrO_2_ [36], have been proposed for memristive memories as well as synaptic type devices. Among them, CeO_2_ has been suggested as the most promising material for renewable nonvolatile memories, as it exhibits a high dielectric constant of ~26 and a high bandgap of 6 eV, which covers the maximum residual bandgap in other oxide materials [37]. The flexible valence states of Ce^3+^ and Ce^4+^ in ceria can create conducting filaments (CFs) containing O_V_s or may disrupt them upon application of a biasing voltage signal. Meanwhile, the versatile asymmetric electrode memristor Ag/CeO_2_/Pt has been widely examined. The resistance state-functioning of an active metal/oxide/inert metal structure was initially tuned by rupturing of the silver CF; moreover, the CF containing defective O_V_s was also comparatively studied [38]. The Ag/CeO_2_/Pt memristor has been demonstrated to be an inherent bipolar resistance switching device with the realization of slow variation in resistance states with low operating voltage, which has enabled artificial synapse simulation [39].

Herein, the main focus areas of research are schematically presented and elaborated. The various types of CeO_2_-based memristors are classified according to filamentary-based mechanisms. Then, the electrode-based (symmetric and asymmetric) structures and their performance are examined. Further, the resistance variation tendency of unipolar, bipolar, and threshold memristors and their role as single- and double-insulating layers (with capping layer) for achieving optimal RS behavior is elaborated. Neuromorphic applications involving spike-based learning, potentiation and depression behaviors, synaptic plasticity, and synaptic weight decay with time modulation are significantly investigated. The extensive review concludes that various CeO_2_-based memristors have demonstrated excellent performance while proving themselves as advanced synaptic devices. Mainly, the focus is on Pavlov’s dog experiment in this study because it represents an advanced synaptic study; accordingly, it has been critically examined here to cover recent advances in CeO_2_-based synaptic devices. Finally, future perspectives for advancement in oxide-based materials toward synaptic trends are suggested and are achievable through the utilization of alternative high-k oxide materials.

## 2. Filamentary Mechanisms in CeO_2_-Based Memristors

The RS mechanisms of various materials have been widely analyzed for elucidating the resistance variation process; however, the resistance switching mechanism in RRAMs is still contentious. Several literature reviews have been presented to understand switching kinetics in different switching materials. Interestingly, no specific switching mechanism assures 100% RS behavior [1,40]. However, the conductive filamentary mechanisms are broadly predictable, whereas major disagreements still prevail concerning microstates, configuration, and shape of the creation and annihilation of the CFs. Initially, one or multiple filaments, i.e., local conducting channels are formed or ruptured when an external stimulus is applied. Some materials may require a forming state with the application of external voltages. In this way, CFs may be created by the occurrence of soft break-up in the insulating layer [41]. Therein, change from a high-resistance state (HRS) to a low-resistance state (LRS) corresponds to the SET process, whereas the converse variation represents the RESET process. Further, under application of a positive voltage at the top electrode (TE), charge carriers trapped by vacancies form a filamentary path, and the device goes into LRS. Conversely, during the reverse mechanism the device will occupy HRS. Akinaga H. stated that the thermally assisted redox reaction with anodization at the metal electrode and oxide-layer interface is responsible for the possible mechanisms [42]. Usually, Joule heating is considered the leading cause of rupturing of conducting paths [43]. However, the typical filamentary mechanism is generally not dependent upon the contact area associated with device electrodes [44,45].

### 2.1. Electrochemical Metallization Filamentary Mechanism

The basic working principle of RRAM cells is the basis for filamentary classification. The filamentary mechanism can be classified as electrochemical metallization mechanism (ECM), valence change mechanism (VCM) [46], and thermochemical mechanism (TCM) [47,48].

#### 2.1.1. Electrochemical Metallization Mechanism

**Figure 1:**
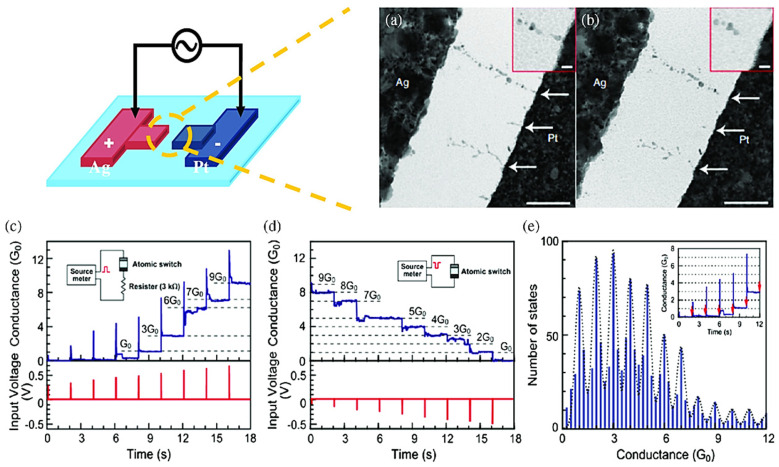
(**a**) TEM result for the cell containing a larger electrode showing a space after the forming process (scale bar: 200 nm). The red color square and zoomed-in image of the filament (indicated with the upper arrows) close to the dielectric/inert electrode edge (scale bar: 20 nm) and (**b**) The red color square and zoomed-in image and TEM image after erasing. Adopted from [52]. Copyright 2012 Nature Publishing Group. Conductance quantization with the synaptic trend of Ta_2_O_5_-based atomic switching: (**c**) observed increment in quantized conductance owing to the cell upon application of positive voltage pulses of width 20 ms during a 2-s interval. The inset shows the controlled-conductance level realized by inserting a series combination with the cell along with a current-controlling resistor with a resistance of 3 kΩ, (**d**) observed decrement in quantized conductance owing to the cell upon application of negative voltage pulses of width 20 ms during a 2-s interval. The input pulses are directly connected to the device, shown in the inset view, and (**e**) The quantized conductance histogram demonstration from measured data with zoomed-in image of conductance against time. Adopted from [53]. Copyright 2012 IOP Publishing.

ECM is often considered to elaborate the working principle of RRAM devices by using metals having high mobility for dynamic electrodes, such as silver (Ag), copper (Cu), and nickel (Ni) [3]. Yang et al. reported the conductive filament mechanism for the Pt/SiO_2_/Ag structure using transmission electron microscopy (TEM) as shown in Figure 1a,b [49]. For new planar architecture formation, conductive channels are not formed within the SiO_2_ layer lying between Ag and Pt electrodes. After application of a positive potential to the Ag electrode, an oxidation reaction occurs (Ag - e → Ag^+^). During the movement of Ag^+^ toward the Pt electrode surface, Ag^+^ loses electrons to undergo reduction and becomes Ag. The Ag provides evidence of Ag conductive filament formation in an RRAM switching layer, thereby exhibiting LRS as illustrated by Figure 1a. By contrast, upon application of a positive voltage at the Pt electrode, the HRS state of the device is observed as shown in Figure 1b, supporting the breakdown of the conducting path.

Yan et al. examined the RS behavior of Pt/SrTiO_3_/Ag RRAM devices using X-ray photoelectron spectroscopy (XPS), investigating the unintended conducting filamentary process related to Ag in memory devices [50]. The prepared device was operated in HRS and LRS states, and complex XPS spectra of Ag and Pt electrodes were obtained. The Ag component always provides evidence of their occurrence between the TE and bottom electrode (BE). Additionally, a slight variation in dispersion intensity may easily be identified at various depths for the SrTiO_3_ film when the device is at LRS. However, in HRS, a slightly insignificant amount of Ag is observed in the SrTiO_3_ film and the Pt thin film. Thus, the role of RS should be implicated in formation and rupture in the established silver filament mechanism. Meanwhile, Tsuruoka et al. have reported the quantized conductance of the Ag/Ta_2_O_5_/Pt structure as presented in Figure 1c–e [51].

#### 2.1.2. Valence Change Mechanism

**Figure 2:**
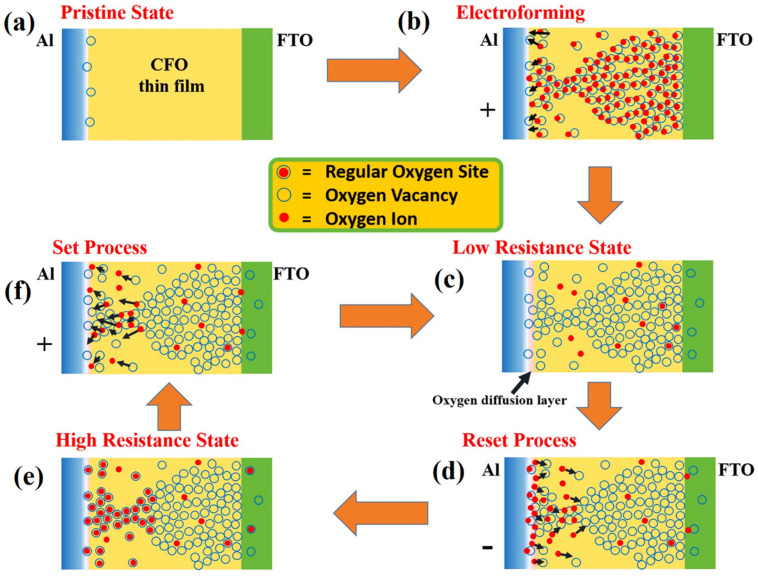
Schematic illustration of (**a**) original state containing electrodes as top Al and bottom FTO, (**b**) electroforming mechanism, (**c**) LRS-containing conducting cobalt ferrite film and oxygen diffusion layer toward the Al electrode, (**d**) reset state showing O^−2^ ions returning and originating from oxygen spreading layer toward the cobalt ferrite film, (**e**,**f**) HRS with the set process. Adopted from [58]. Copyright 2017 Nature Publishing Group.

VCM and ECM exhibit the same trends with respect to electrochemical reaction and ion mobility mechanisms. ECM is often considered as an electrochemical reaction involving active metals, whereas VCM is also electrochemical reaction, though it is based on O_V_s in oxide materials. Generally, the device resistance change is significantly affected by oxygen-related defects in electrochemical reactions. The extensive literature has reported that the transition between HRS and LRS corresponding to the desired memory device (such as RRAM) is caused by the damage or formation of O_V_ filaments [3,54,55,56,57]. The steps involved in the VCM process are presented in Figure 2a–f. When positive biasing is applied at an inert TE, the oxide insulating layer becomes active in LRS; O^2−^ changes their original position, and the O_V_s alternatively produce a conductive path along with drifting toward the anode/oxide-layer interface. In this way, a conductive path containing O_V_s is eventually created, increasing the conductivity owing to the functional layer film.

Conversely, upon application of the reverse voltage, the formed conductive channels are gradually destroyed, and the RRAM device turns transitions to the HRS. Consequently, O^2−^/O_V_s may be responsible for developing and rupturing conducting channels in RRAMs. Szot et al. reported the locality in conductivity related to SrTiO_3_ films and discussed it in detail by employing atomic force microscopy with a conducting tip, observing that SrTiO_3_ films may demonstrate bistable conductance switching between nonmetallic and metallic behavior upon application of an electrical field [56]. In addition, a later study reported in favor of bipolar resistance switching behavior, resulting in an ideal conductive channel among the Pt tops upon being influenced by Ta-rich clusters belonging to asymmetric TaO_x_, which were directly perceived by an in-situ detection approach [58]. Notably, VCM generates the O_V_ – O^2−^ anti-Frenkel pairing. However, the formation of O_V_ – O^2−^ anti-Frenkel pairing has not been reported in the literature [59,60]. The formation of O_V_ – O^2−^ anti-Frenkel pairing would cause instability and relaxation [47].

#### 2.1.3. Thermochemical Mechanism

**Figure 3:**
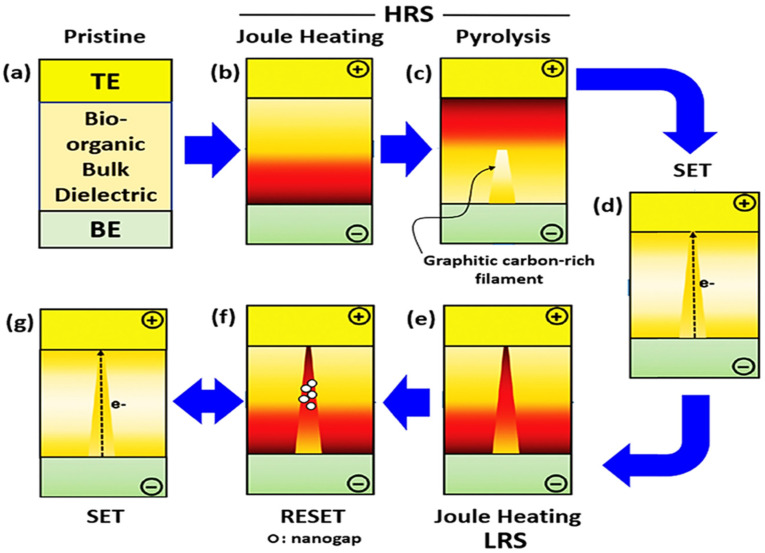
Schematic depiction of thermochemical process showing the RS of (**a**) pristine state, HRS under (**b**) Joule heating and (**c**) pyrolysis, (**d**) set process associated with conducting channel creation, LRS under (**e**) Joule heating, (**f**) reset process associated with CF breakdown, and (**g**) reversion to the set process. Adopted from [67]. Copyright 2018 John Wiley & Sons, Ltd.

RRAM cells commonly show resistance variation with the application of an electric field and Joule heating-based O^2−^ reactions associated with drifting that dominate the operation mechanism [61]. In these RRAM cells, the forming process is attributable to thermal rupturing within the device medium and the subsequent formation of CFs. By contrast, the reset process corresponds to thermal melting of the dominant CFs [62,63]. Moreover, unipolar/bipolar modes are considered more effective in RRAM cells owing to the free Joule heating development without a polarity effect. The different steps involved in TCM are presented in Figure 3a–g. The nonpolar switching in resistance change has exhibited a particular trend in some previous studies. Jung et al. demonstrated a Pt/NiO/Pt memory structure and discussed temperature-dependent resistance change in switching dynamics in NiO films, revealing the corresponding OFF-state current evaluated with respect to temperature and defect patterns.

The Pt/TiO_2_/Pt memory filamentary arrangement is openly determined during resistance variation by employing high-resolution TEM [64]. In situ voltage–current (V–I) curves at a lower temperature (130 K) evidenced the creation and rupturing of filaments owing to TinO_2n-1_. Upon increasing O_V_ density, triangular-type filaments containing TinO_2n-1_ of the Magneli phase were slowly formed in the set process. This result was obtained using the Fast Fourier transform, from which the diffraction pattern of Ti_4_O_7_ was simulated. When the thermal influence is considered with a reversed external field, TinO_2n-1_ is converted back into TiO_2_ within the prevailing triangular state of the filament, where it rearranges the O^2−^ concentration. Hence, Joule heating always plays a pivotal role in rupturing the CFs, which is broadly recognized. However, there is still no prominent experimental confirmation verifying the factor accountable for the formation of CFs. Several factors cause the creation and disruption of CFs, including electrode range, reaction, and switching layer thickness [65]. Additionally, various materials show a variety of storage mechanisms in memory devices. The above summary is only for filament mechanisms; however, specific mechanisms are still under discussion [66]. Because the physical mechanism for the RS phenomenon has not yet been elucidated, it should be focused on in future investigations [47].

## 3. Unipolar and Bipolar CeO_2_-Based Memristors

**Figure 4:**
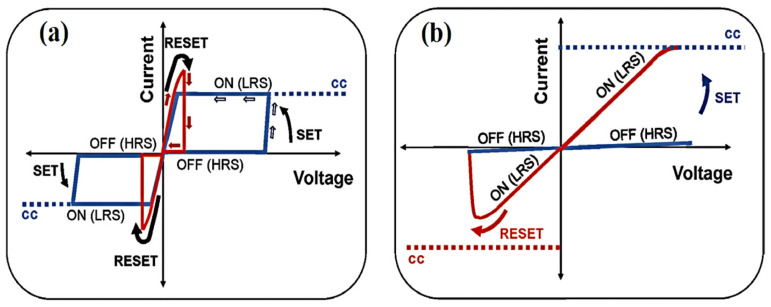
Schematic of (I–V) switching performance of memristors for (**a**) unipolar type, (**b**) bipolar type, and for compliance current (CC). Adopted from [69]. Copyright 2016 Walter de Gruyter GmbH.

RRAM is a nonvolatile memory, showing two logical resistance states of the switching channel. It characteristically involves an insulating or semiconducting layer, mainly composed of high-k oxide nanomaterials sandwiched between two electrodes as shown in Figure 4a. These memory devices commonly exhibit hysteresis performance with current and voltage characteristics (i.e., negative differential resistance at a specific applied potential). In the SET state, when the applied voltage and current approach a threshold level, RRAM cells switch from the HRS (OFF-state) to the LRS (ON-state). Similarly, upon application of a specific voltage, the RRAM cells return to their OFF-state when the prevailing tendency is removed. In nonpolar RRAM, as shown in Figure 4b, the SET and RESET modes work with unidirectional applied voltage. By contrast, in bipolar RRAM, the resistance may vary step-wise, showing two distinct values; however, some devices exhibit gradual resistance variation depending upon the previous path of charges [68].

**Figure 5:**
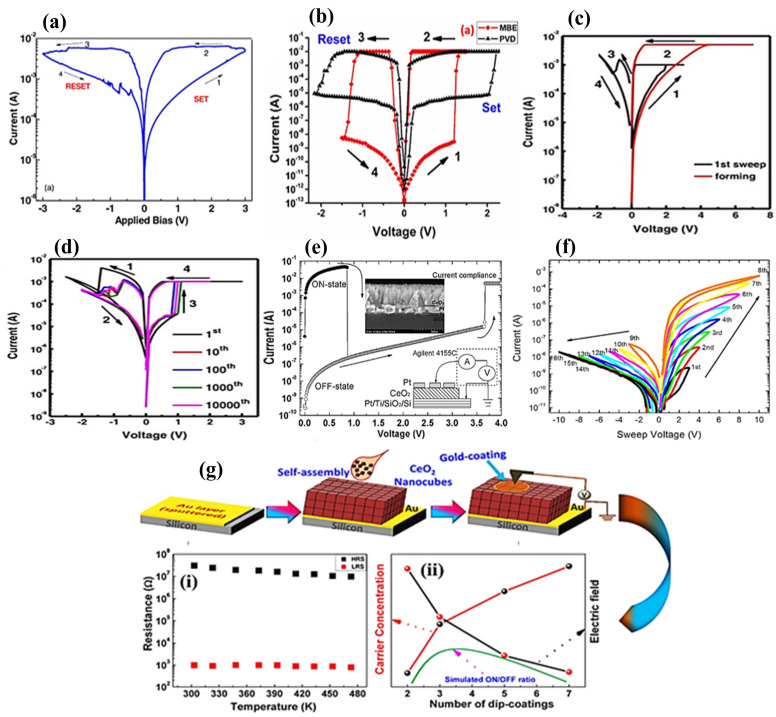
(**a**) Typical semilogarithmic I–V curves of the Au/CeO_2_/ITO device. Adopted from [70]. Copyright 2012 IOP Publishing. (**b**) Typical DC sweep I–V characteristics of the Al/CeO_2_/Au cell. The order of the arrows discloses the sweeping path with numbers. The value of 1 μm indicates the diameter of each device lying between Au (30 nm), CeO_x_ (13 nm), and Au (30 nm). Adopted from [15]. Copyright 2015 American Institute of Physics. Typical bipolar-mode I–V curves indicating the RS trend for the Zr/CeO_x_/Pt structure containing CeO_x_ layers with different thicknesses: (**c**) 25 and (**d**) 14 nm. Adopted from [16], Copyright 2014 Springer Nature. (**e**) Typical I–V graphs for the Pt/CeO_2_/Pt device at the semilogarithmic scale and inset showing the scanning electron microscopy (SEM) image of the CeO_2_/Pt/Ti/SiO_2_ construction. Adopted from [17]. Copyright 2008 Elsevier B.V. (**f**) Semilogarithmic I–V curves under voltage sweep. Adopted from [18]. Copyright 2017 IOP Publishing. (**g**) Schematic depiction of self-assembled CeO_2_ nano cubes for the functioning of the RS device with the I–V measurement scheme, (**i**) temperature dependence of R_OFF_ and R_ON_ for the device to indicate semiconducting performance. The inset shows the OFF-state Arrhenius plot, (**ii**) Change in carrier density under an electric field, and simulation of OFF/ON ratio under variation in film thickness. Adopted from [71]. Copyright 2013 American Chemical Society.

**Figure 6:**
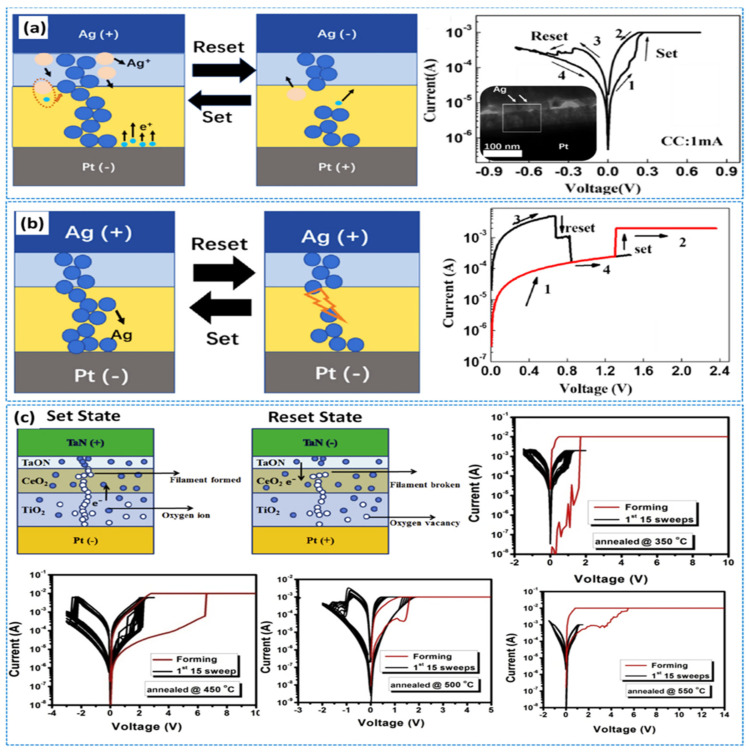
(**a**) Demonstration of electrochemical process for the creation and rupture of conducting silver filaments corresponding to log (I)–V characterization for the Ag/Ti/CeO_2_/Pt structure. (**b**) Schematic depiction of the URS for the Ag/Ti/CeO_2_/Pt device, with I–V graphs for this memory device indicating unipolar operation trend. Adopted from [32]. Copyright 2020 Elsevier B.V. (**c**) Schematic illustration of the conducting channels in a TaN/CeO_2_/TiO_2_/Pt cell with corresponding graphs showing ideal BRS behavior at annealing temperatures of 350 °C, 450 °C, 500 °C, and 550 °C. Adopted from [76]. Copyright 2017 Elsevier B.V.

Sun et al. reported the bipolar switching mechanism in the Al/CeO_x_/Pt cell associated with multilevel resistance variation through the creation and rupturing of CFs containing O_V_s by valence change from Ce^3+^ to Ce^4+^ cations [14,18]. Many researchers have demonstrated a digital-type resistance variation for RRAM applications by utilizing the CeO_2_ switching layer [14,15,16,70] through typical I–V curves for the RS trend involving the TE area (0.25 mm^2^), employing a sweeping voltage at 300 K. Figure 5a depicts the statistics of the configuration for SET and RESET voltages. Younis et al. presented the switching operation evaluated through O_V_s in the Au/CeO_2_/ITO structure, as shown in Figure 5b [70]. In addition, Hsieh et al. [15] and Ismail et al. [16] have discussed the bipolar switching behavior of Al/CeO_2_/Au and Zr/CeO_x_/Pt structures, respectively, as shown in Figure 6c–e. Further, Lin et al. reported the same trend in the CeO_2_ conductive layer for Pt/CeO_2_/Pt structures as shown in Figure 5g [18]. Despite this, the analog switching of a CeO_2_ layer is still being researched [18]. An analog memristive switching along with an excellent resistance variation by a few orders of magnitude in a Pt/CeO_2_/Pt memristive nature cell has been investigated. Pt/CeO_2_/Pt possessed symmetric Pt electrodes (both top and bottom); therefore, it showed significant polarity-dependent and asymmetric diode RS mode as depicted Figure 5f [18]. In Younis et al.’s study [71], bipolar reversible RS behavior was observed when a self-assembled CeO_2_ nanocube-derivative layer of ~162-nm thickness was decorated on the Au-coated Si-substrate. Compared to the thin CeO_2_ layer, the bulky and uneven grains attributed to CeO_2_-dependent nano cubes exhibit extra O_V_s, thereby leading to improved performance in the form of an excellent OFF/ON ratio, small forming potential with a reasonable resistance variation process. Further, the regular cubic-type crystals occupying increased O_V_s at the surfaces rather than their counterparts contribute to reducing the set voltage while rearranging themselves in forming regular percolation paths, as it is deficient in bulk-type thin films. In addition, a CF model containing O_V_s with perfect distribution has been suggested to describe the RS process.

Bipolar resistive switching (BRS) and unipolar resistive switching (URS) have been achieved in a double-layer Ag/Ti/CeO_2_/Pt structure under different sweep rates along with CC. In BRS, the RS trend depends upon ECM, whereas TCM is dominated by URS functioning (Figure 6a,b). No forming process and small RS voltage make the device more attractive [39]. Here, a sweeping voltage mode associated with a sweeping potential (0.005 V) at a rate of 0.09 V/s is followed. Further, sweeping voltage operation in the order of 0 V → 1 V → −1 V → 0 V favors bipolar behavior of the device (as in Figure 6a). The device shows the HRS of 2800 Ω with a sweeping potential of 0–1 V. Then, the resistance state changes to LRS, exhibiting 270 Ω at 0.3 V. In this case, the CC (1 mA) is observed. Negative biasing is dominated by CF [72]. In addition, I–V curves exploring the space-charge-limited current (SCLC) obey Ohm’s law (I ∝ V) and Child’s law (I ∝ V^3/2^). Furthermore, to confirm the bipolar switching operation, double-log I–V curves are also depicted. However, the primary defect lying in the device is its deviation from the SCLC mechanism during RS functioning [39]. To establish the repeatability and reproducibility of bipolar switching behavior, a total of 120 cycles were executed. During this experiment, the SET voltage was maintained at 0.3 V, whereas the RESET voltage spanned from −0.3 to −0.6 V. Notably, no compliance current (CC) was attained during the RESET phase, thereby enabling the realization of maximum achievable current levels.

By contrast, a current of 2 mA was set during SET state, whereby URS behavior was observed as shown in Figure 6b. Further, the current increased to 1.4 V and the resistance states changed to SET. Afterward, at a positive voltage (without limiting current), the device changed from LRS to HRS operating at a potential of 0.7 V (but without the forming step). TCM [73,74] was followed to explain the URS trend associated with the CFs during the LRS state. The local current density in the conductive channel becomes very large, resulting in Joule heating, which sharply raises the temperature of the conductive filament. Then, the conducting channel breaks while returning the device to a high resistance. Further, bipolar RS characteristics were explored in a bilayer TaN/CeO_2_/TiO_2_/Pt structure. The set voltage was found to be dominated by CeO_x_ layer thickness, indicating the switching process to be an electric field-induced mechanism. A CeO_2_/TiO_2_ ultrathin film was deposited by radio-frequency magnetron sputtering with an Ar/O_2_ mixture, followed by annealing at different temperatures (350 °C, 450 °C, 500 °C, and 550 °C. The annealed samples had decreased roughness and set/reset voltages (~1.1 and ~1 V) at 500 ºC as shown in Figure 6c. Here, the TiN/CeO_x_ interface performed the principal role of controlling O_V_ defects, thus controlling the creation/deforming of conductive paths. This device also showed cycling endurance with an HRS-to-LRS ratio of 10^5^ with excellent performance under retention/stress test (10^4^ s). Cycling endurance with data-retention performance was acceptable [75]. The coexisting RS behaviors were comprehensively characterized for the Ti/CeO_2_/Al/CeO_2_/Pt device. Similar SET and RESET voltages and similar current levels with the exact current compliance in the SET process validated that the device operation was very simple. Both coexisting RS trends were identical during the LRS and HRS modes. However, the switching uniqueness among both modes was investigated in the CeO_2_ devices involving an Al capping layer. The BRS characteristics of lower voltage with optimum operation window in CeO_2_/TiO_2_-based resistive memories with multilevel switching were analyzed. The CeO_2_/TiO_2_ ultrathin film was decorated through a radio-frequency (RF) magnetron sputtering route in an Ar/O_2_ environment, followed by annealing at various temperatures. The annealing process considerably reduced the unevenness and set/reset window of ~1.1/~1 V at 500 °C. A more negative value of Gibbs free energy owing to TiO_2_ compared to CeO_2_ leads to a more straightforward reoxidation process for filamentary oxygen exchange with a tantalum nitride electrode. In this respect, cumulative probability distribution of fundamental memory parameters has exhibited reasonable configuration of the SET/RESET potential. The said device offered outstanding endurance properties, such as DC switching operation at >2500 cycles, data retention >10^5^ s, and a resistance ratio of >10^2^ at a temperature of 500 °C [76].

**Figure 7:**
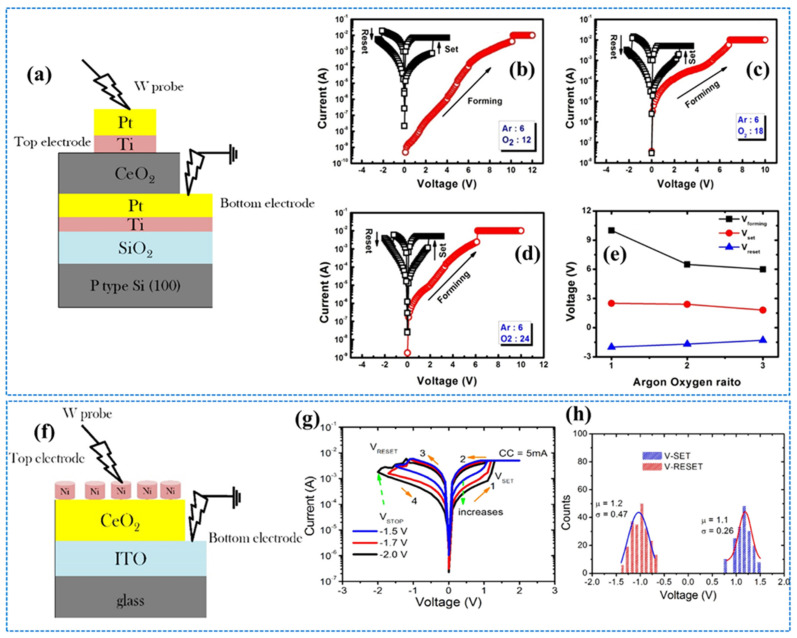
(**a**) Schematic depiction of the Ti/CeO_2−x_/Pt device, indicating the addition of an insulating layer between two electrodes. Arrows display the I–V measurements. Typical I–V curves show the initial electroforming process with initial sweeping for a bipolar RS trend in Ti/CeO_2−x_/Pt cells containing different Ar/O_2_ amounts for (**b**) specimen A, (**c**) specimen B, (**d**) specimen C, and (**e**) forming voltage with SET/RESET processes involving the Ar/O_2_ ratio in the CeO_2_ layer depositing chamber. Adopted from [77]. Copyright 2018 Springer Nature. (**f**) Schematic illustration of the Ni/CeO_2_/ITO/glass structures, (**g**) I–V curves for the Ni/CeO_2−x_/ITO/glass device measured at different RESET-stop voltages ranging from −1.5 to −2.0 V, and (**h**) statistical distribution of the SET and RESET voltages. Adopted from [78]. Copyright 2019 Elsevier B.V.

The bipolar RS behavior of CeO_2−x_ ultrathin layers with an ultrathin TiO contacting layer under an Ar/O_2_ mixture in the chamber is shown in Figure 7a. The titanium oxide layer with an appropriate Ar/O_2_ mixture ratio is considered more suitable for the stability as well as uniformity of the RS mechanism. Moreover, a CeO_2−x_ film deposited in an Ar/O_2_ (6:24 ratio) environment exhibited outstanding RS behavior owing to the decisive role of existing O_V_s, as shown in Figure 7b–e. In the study, the SCLC with Schottky emission was the dominant transportation in the stronger-field segment. Ideal swapping homogeneity (ON/OFF ratio of 10^2^), device-to-device uniformity with the best DC endurance of >500 cycles, retention performance of 10^4^ s validates it as a suitable nonvolatile RRAM device [77]. The coexistence of both cerium ions (i.e., Ce^+3^ and Ce^+2^) in the oxygen defective CeO_2-x_ layers decorated over an ITO/glass substrate by RF magnetron sputtering was also studied. Furthermore, the Ni/CeO_2−x_/ITO/glass structure displayed a multilevel RS trend as shown in Figure 7f, which was achieved by controlling the RESET-stop potential in the adjustment of SET-CC. The reproducible switching effect and excellent endurance of ~10^3^ DC cycles with a retention time of >10^4^ s was observed. I–V characteristics revealed the Schottky emission transportation mechanism at RESET-stop potentials ranging from ~1.5 to ~2.0 V as shown in Figure 7g,h. These results favor the said device to be beneficial for multilevel information-storage applications [78]. The Pt/CeO_2_/Pt device showed the necessary forming process under application of a potential of 9 V and a current of 5 mA (at CC). Semilogarithmic I–V curves for the device were plotted under unipolar voltage sweeps and ON/OFF state, where the resistance ratio could approach five orders of magnitude. The ON/OFF states were observed with a stability of 10^4^ s at a potential of 0.3 V, showing exceptional nondestructive readout characteristics. Furthermore, the symmetric electrode structure of the same device exhibited RS functioning free from biasing in the sense that both positive and negative potentials may switch ON/OFF states to OFF/ON states (not shown here). In this study, the OFF state was reported to be less stable than the ON state.

## 4. Threshold CeO_2_-Based Memristors

**Figure 8:**
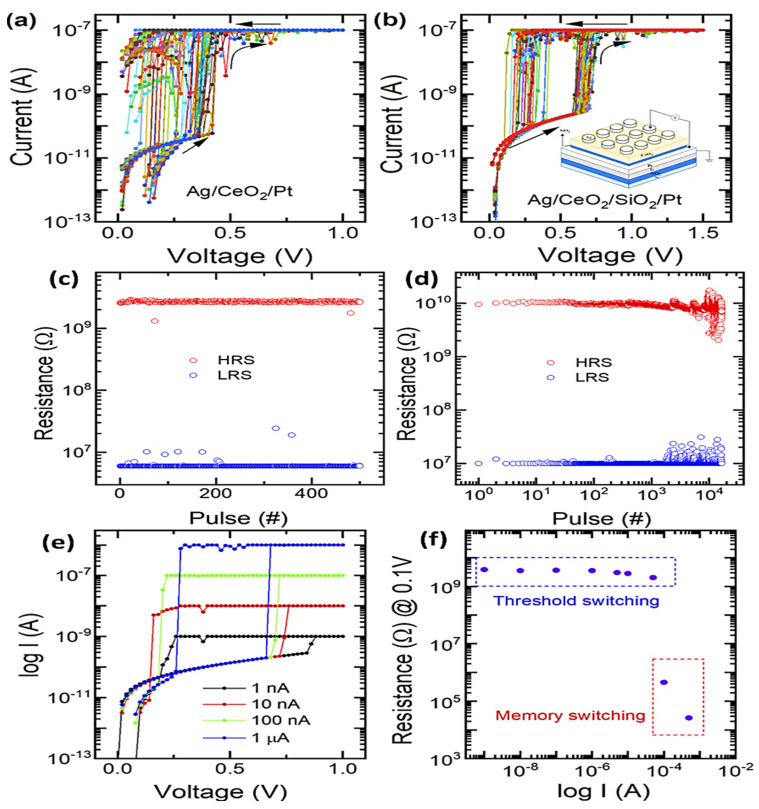
I–V characteristics displaying threshold behavior for (**a**) Ag/CeO_2_/Pt cells and (**b**) Ag/CeO_2_/SiO_2_/Pt cells. Endurance performance of (**c**) Ag/CeO_2_/Pt device and (**d**) Ag/CeO_2_/SiO_2_/Pt device under pulse operation mode. Endurance of the Ag/CeO_2_/SiO_2_/Pt device, (**e**) threshold switching behavior of the Ag/CeO_2_/SiO_2_/Pt device with variable CC, and (**f**) increment in ON-state resistance along with CC. Adapted from [79] Copyright 2022 American Institute of Physics.

Threshold-switching-type memristors have become attractive for selectors of crossbar memory structures. The threshold switching memristor involving a single layer of CeO_2_ transforms into a new memory over repeated cycles caused by an overgrowth of Ag filament, thereby changing the device to LRS. Hence, optimal threshold switching was suggested in the aforementioned diffusive memristors by taking CeO_2_ as a switching layer composed of Ag filaments (with low voltage) combined with a SiO_2_ diffusion barrier layer in controlling the filament formation to achieve an advanced memory device. In this respect, an amorphous-type SiO_2_ layer was placed underneath the CeO_2_ layer, behaving as an obstacle for the overgrowth of silver metal filaments and thereby enhancing the device stability. This tailored device has exhibited a forming-free volatile threshold switching nature (with 1 pA OFF-state current) and excellent selectivity of 10^4^ with tuned uniformity and endurance characteristics. Moreover, Ag/CeO_2_/SiO_2_/Pt has shown outstanding direct current for 10^3^ I–V cycles with low density at switching voltages. Because of such results, the implementation of CeO_2_-based threshold RRAMs as the best selectors is proposed when crossbar memories are adopted [79]. The I–V characteristics of both Ag/CeO_2_/Pt cell and Ag/CeO_2_/SiO_2_/Pt cells under 50 consecutive cycles are presented in Figure 8a,b, and the endurance performance of the two devices under pulse operation is shown in Figure 8c,d. The Ag/CeO_2_/SiO_2_/Pt structure exhibited stability in endurance performance for 104 cycles, sustaining an on/off ratio of 10^3^. A pulse rate of 1 with 0.6 V and duration of 0.64 ms was applied for switching operation in the abovementioned devices by incorporating a read pulse of 0.2 V for a duration of 0.64 ms as shown in Figure 8e. Threshold switching of the Ag/CeO_2_/SiO_2_/Pt structure occurs under variable CC. However, an increment in the ON-state with CC is also investigated, as shown in Figure 8f.

## 5. Electrode-Based CeO_2_ Memory Devices

### 5.1. Symmetric Electrode-Based CeO_2_ Memristors

**Figure 9:**
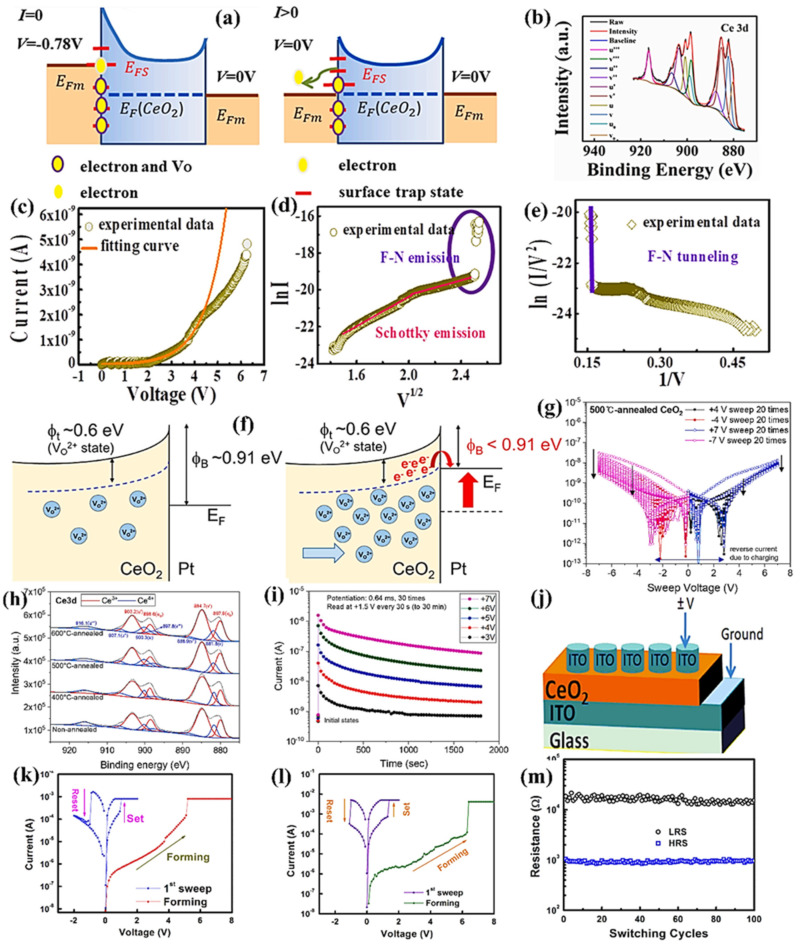
(**a**) Energy band diagram for semiconducting metal interface and surface states at different reverse biases. (**b**) Ce 3d deconvolution XPS results for 30-s sputtering sample. I–V trends for samples with fitting curves: (**c**) Schottky emission fitting curves, (**d**) Schottky emission fitting lines, (**e**) F–N tunneling fitting lines. Adopted from [81]. Copyright 2022 Elsevier B.V. (**f**) Schematic display of Schottky barrier-height tunning occurring at the CeO_2_/Pt junction by O_V_ redistribution when positive voltages are applied to the top Pt- electrode, extracting O_V_s near the bottom contact. (**g**) I–V curves of the reference device under annealing of Pt/SiO_2_/CeO_2_/SiO_2_/Pt cell at 500 °C. (**h**) XPS spectra of the Ce 3d levels for nonannealing and at 400 °C, 500 °C, and 600 °C annealing for Pt/CeO_2_/Pt memristors. (**i**) Retention performance of Pt/CeO_2_/Pt memristors at 500 °C annealing upon repeated application of +3–+7 V pulses with 30 repetitions and measurement of current during 1800 s. Adopted from [26] Copyright 2022. Elsevier B.V. (**j**) Arrangement schematic of the ITO/CeO_2_/ITO device with I–V curves showing the starting forming mechanism with prior sweeping of the BRS trend for ITO/CeO_2_/ITO devices containing CeO_2_-layers with different thicknesses: (**k**) 15 and (**l**) 20 nm. (**m**) Endurance performance of the ITO/CeO_2_/ITO device. Adopted from [80] Copyright 2015 Elsevier B.V.

Memristors containing both electrodes (the same in nature) are regarded as symmetric electrode-based memristors. The widely used CeO_2_ memristors of this type are focused on in this section. In this respect, being symmetric electrode-based, spin-coated Pt/CeO_2-x_/Pt with a polycrystalline phase and mixed with fluorite cubic structures having 40-nm thickness of Pt layer are deposited through DC magnetron sputtering. The energy bands attributed to semiconductor–metal contact involving surface states with different reverse biases and the XPS spectrum during 30-s sputtering revealing the binding energy are shown in Figure 9a,b. Two fundamental damages occur in the CeO_2-x_ film of Pt/CeO_2-x_/Pt cell during electroforming phenomena. Surface defects are generated in Pt/CeO_2-x_ at the TE contact, which provides the source to create positive charge carriers into CeO_2-x_ film. During the set process, electron tunneling is accompanied by O_V_ ionization in Pt/CeO_2-x_ at the TE junction. The various trapping states occurring at the metal–semiconductor junction during metal electrode fabrication is evaluated in the form of surface damage. The repeated potential field owing to the crystal is disturbed at the surface, resulting in extra energy levels. Every surface atom is ascribed to a single surface energy level existing in disallowed states. Therefore, ionized O_V_s would be allowed to be dispersed at energy levels, owing to which electrons are confined in trapping sites. Moreover, the greater radius of Ce^3+^ than that of Ce^4+^ may produce an extended lattice and distortion. The distortion in lattice and grain boundaries often breaks up the lattice order. Consequently, the ionized O_V_s drift from the TE toward the BE, and many O_V_s are detected as electron-trapping sites in the bulk state. The I–V characteristics, Schottky emission fitting curves, and F-N tunneling fitting lines are displayed in Figure 9c. Further, a schematic illustration of the Schottky barrier altitude optimization occurring at the bottom CeO_2_/Pt contact is shown in Figure 9f. It results from O_V_ redistribution with initial and final application of positive voltage existing at the top Pt electrode when O_V_s are transferred toward the bottom contact as shown in Figure 9f [32]. The Pt/CeO_2_/Pt memristors were prepared by depositing a 50-nm-thick CeO_2_ resistance-based layer on a Pt electrode on the Ti/SiO_2_/Si substrate through RF magnetron sputtering when the CeO_2_ target was selected with an Ar medium. Subsequently, an annealing process (400 °C–600 °C for 1 h) was also performed in ambient air. A platinum TE having a diameter of 100 μm was deposited by electron beam evaporation.

Further, the Pt/CeO_2_/Pt structure exhibited various current values with the alteration of positive voltage sweeps, which gave higher values than negative voltage sweeps. The I–V curves of the reference device under annealing at 500 °C with 20 repeated voltage sweeps are shown in Figure 9g; the I–V curves reveal that the device is electrically nonsymmetric because of the bottom Pt/CeO_2_ junction formation, as CeO_2_ is deposited through sputtering. By contrast, the top Pt/CeO_2_ junction is established through Pt electrode deposition on the CeO_2_ layer through evaporation. In addition, the annealing process is performed after CeO_2_ film deposition, leading to a bottom interface, whereas the top interface does not experience annealing. Consequently, asymmetric electrical characteristics are achieved [26]. RS characteristics have also been exhibited by completely transparent ITO/CeO_2_/ITO devices. The physical schematics with endurance performance of such a device are presented in Figure 9j,m. CeO_2_ ultrathin films containing 15–25 nm thin layers are decorated by RF magnetron sputtering. Additionally, a CeO_2_ ceramic target is decorated over ITO coated with 200-nm-thick glass substrate in an Ar–O_2_ (ratio of 20:10) environment as illustrated in Figure 9k–m. For RS results, a 75-nm-thick of ITO TE with a diameter of 150 μm is deposited by RF magnetron sputtering. When ITO is deposited, the pressure in the chamber is controlled to a value of 10 m Torr under Ar gas atmosphere with 100-W sputtering power [80].

### 5.2. Asymmetric Electrode-Based CeO_2_ Memristors

Asymmetric electrode-based memristors contain different TE and BE and show comparatively superior characteristics than symmetric electrode-based devices. Therefore, CeO_2_-based asymmetric memristors are discussed in detail in this section. The Ag/CeO_2_/Pt device is prepared by providing a high-vacuum environment during magnetron sputtering and a coating structure exhibiting asymmetric electrode behavior. Ceria layers having a thickness of 27 nm are decorated over a Pt/Ti/SiO_2_/Si substrate by RF magnetron sputtering. Then, a silver TE with a diameter of 200 μm is deposited on the as-prepared film with a shadow mask. The ceria thin film exhibits good compactness, which is confirmed by its average roughness of 0.38 nm. This ceria film shows sufficient crystallinity and pure phase [32]. Meanwhile, an asymmetric Ag/CeO_2_/Pt device is favored according to Wang et al. Ag filaments incorporated into the ceria conducting layer are vertically stacked in the order of BE to TE [82], which significantly favors Ag^+^ ions with high mobility compared to electrons because the ceria lattice provides a fast diffusion channel for Ag^+^ ions [32].

**Figure 10:**
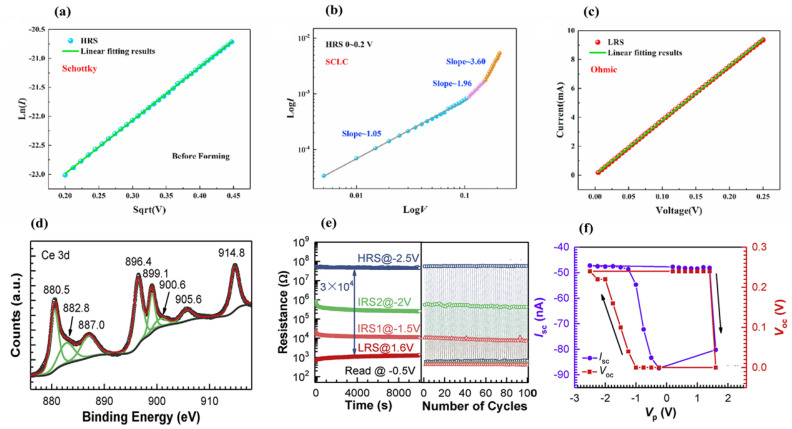
Fitting results of I–V curves at three levels: (**a**) prior to forming, (**b**) transition in resistance states, and (**c**) at LRS. Adopted from [39]. Copyright Elsevier B.V. (**d**) Ce 3d level XPS spectra of the CeO_2_/NSTO device. (**e**) Retention/endurance efficiency at LRS, IRS1, IRS2, and during illumination. (**f**) I_sc_ and V_oc_ of the device during illumination at various applied voltage pulses. Adopted from [83]. Copyright 2019 Elsevier B.V.

In the Ag/CeO_2_/Pt memristive structure, the variation in the corresponding rate and voltage range of the device gradually changes the resistance. It is controllable through the creation and rupture of silver filaments; the conduction phenomena corresponding to each state are also investigated. The semilogarithmic I–V curves, endurance performance under 100 repetitions, retention characteristics depending on ambient temperatures, fitting results for three levels before the forming process, and transition of states are shown in Figure 10a–f. The resistance transition of the memristor is stable during 100 voltage sweeps; thus, the resistance states are retained for 5 × 10^4^ s [32]. In another study, epitaxial CeO_2_ films were synthesized via pulsed laser deposition. In this structure, 0.7-wt.% Nb-doped SrTiO_3_ single crystals with sizes of 3 and 0.5 mm were used for the deposition substrate. The Pt TE was decorated over a CeO_2_ layer by DC magnetron sputtering along with a shadow mask having many holes with an aperture of 0.2 mm. Furthermore, the indium BE was tarnished on NSTO substrates for forming an ohmic contact. This device exhibited outstanding RS behavior with an RS ratio of 3 × 10^4^. A significant photoresponse was detected in this device when it was illuminated with a 405-nm laser beam, which facilitated fine-switching operation at HRS. In addition, both light-controlled RS associated with voltage-controlled photo-responsiveness were evaluated in the device. These RS and photoresponse properties are ascribed to the Schottky barrier transport in the Pt/CeO_2_ junction, whereas electron trapping/de-trapping occurs because of O_V_ insertion into the interface. The main focus is to explain the binding energy levels using the XPS spectra of CeO_2_/NSTO at the Ce 3d level. Furthermore, I–V characteristics under darkness and 405-nm illumination and retention behavior at LRS, IRS1, IRS2, and HRS in the dark were obtained. The retention and endurance at LRS, IRS1, IRS2, and during illumination; resistance levels during dark and illumination under different voltage pulses; short-circuit current; and open-circuit voltage during illumination with the provision of voltage pulses are presented in Figure 10d,e. Because of such peculiarities, they may afford potential applications for different levels of RS memories along with various functions in photoelectric sensors [83].

To add, a layer 10-nm-thick CeO_2_ film is decorated over the Pt BE of the Pt/Ti/SiO_2_/Si substrate, which is synthesized by RF magnetron sputtering. Further, a 5-nm-thick ZnO thin film is used as a second dielectric layer that is decorated over CeO_2_/Pt/Ti/SiO_2_/Si with the same parameters following the CeO_2_ layer deposition. The final structure is the double-layered structure of the M/ZnO/CeO_2_/Pt device, for which alternative electrodes such as Ti, TaN, and TiN are deposited at normal temperature. Here, magnetron sputtering is adopted with a metal shadow mask to be circular as TEs (100-nm thick) and with a diameter of 150 μm. Further, the effect of Gibbs free energy on oxidation with the electronegativity of the TE material is also analyzed. It has been observed that decreased operational voltages may occur incrementally because of the work function gap between the TE and BE. Interfacial contact between the TE and the oxide layer (ZnO) influences the RS phenomenon. The forming and subsequent RS functioning of various double-layered structures, such as M/ZnO/CeO_2_/Pt, TaN/ZnO/CeO_2_/Pt, TiN/ZnO/CeO_2_/Pt, Ti/ZnO/CeO_2_/Pt, and Ni/ZnO/CeO_2_/Pt, are elaborated in this study. Statistical information related to SET and RESET voltages, resistance states, and switching ratio of the M/ZnO/CeO_2_/Pt devices are presented as well. The devices containing TaN (TE) have exhibited excellent cycling uniformity corresponding to switching parameters, SET and RESET voltages, HRS and LRS resistances, and remarkable endurance properties (>1000 cycles) with LTS > 10^5^ s [84]. Moreover, the endurance performance of TaN-TE, TiN-TE, and Ni-TE with a schematic demonstration revealing he conduction phenomena in TaN/ZnO/CeO_2_/Pt devices is provided. In addition, pristine resistance trends showing the interfacial barriers and LRS at the SET voltage and HRS at the RESET voltage are considered in this study.

## 6. Single-Layer and Bilayer CeO_2_ with Capping Layer

**Figure 11:**
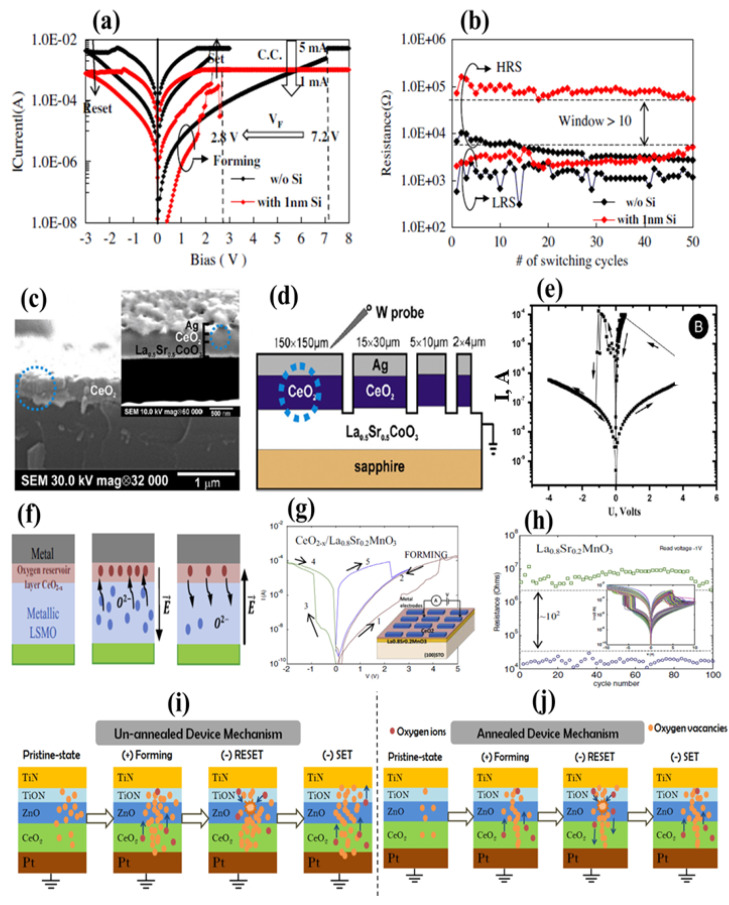
(**a**) Typical RS performance of the W/CeO_2_ (20 nm)/TiN and W/CeO_2_ (20 nm)/Si (1 nm)/TiN devices, (**b**) comparison of HRS and LRS during 50 cycles for RRAMs with and without 1-nm Si buffer layer. Device stability window without and with Si (distinguishable window greater than 10). Adopted from [87]. Copyright 2012 Elsevier B.V. (**c**) SEM photographs, (**d**) schematic view of Ag/CeO_2_/La_0_._5_Sr_0_._5_CoO_3_ decorated over sapphire substrate and analyzed by the focused ion beam technique, and (**e**) typical I–V measurements under electrical forming for various cell sizes. Adopted from [88]. Copyright 2014 Elsevier B.V. (**f**) Schematic demonstration of oxygen (O^2−^) ion movement in the metal/CeO_2-x_/La_0_._8_Sr_0_._2_MnO_3_ cell. (**g**) Graphical representation of the probe scanning over Ag electrodes for CeO_2_/La_0_._8_Sr_0_._2_MnO_3_, the forming step is needed to induce the BRS, which is maintained at low voltage operation. (**h**) Endurance cycling measurement of LSMO structures. The resistance ratio covering the range of 10^2^–10^3^ with a −1V read voltage is measured. The supplementary view representing successive memory functioning in 100 cycles is observed. Adopted from [91]. Copyright 2015 Elsevier B.V. Schematic representation of the RS mechanism (**i**) without annealing and (**j**) annealing treatment in TiN/ZnO/CeO_2-x_/Pt structures [92]. Copyright 2018 Elsevier B.V.

Most previous studies have reported that the RS behavior of CeO_2_ ultrathin films often requires a forming voltage [17]. On the contrary, a study has reported that Ce-silicate can also be structured at the CeO_x_/Si junction [85]. In this way, creating Ce-silicate may increase the density of O_V_s at the contact area [86]. Hence, Si (buffer layer) plays a role at the contact point and in the appearance of O_V_s, thereby facilitating the modulation of the forming process, possibly by CeO_2_ for achieving reversible RS. Additionally, the probability of the suppressed forming process is expected to be assured through interface engineering. However, inserting a Si buffer layer at the bottom contact would form an RRAM cell having the W/CeO_2_/Si/TiN structure with a small forming voltage and CC, offering a reasonable window and endurance features compared to those devices (without Si). It is expected to improve the development of Ce-silicate with the appearance of O_V_s at the interfaces caused by the dynamic production of conducting channels toward the forming process. Figure 11a,b reveals the RS characteristics of the W/CeO_2_/TiN and W/CeO_2_/Si/TiN devices. These devices were operated on similar grounds, and the sweeping voltage order was from 0→10, 0→−3→0, and 0→3→0 V in distinguishing the forming and set/reset processes, respectively. With the insertion of Si, the forming voltage was reduced from 7.2 to 2.8 V, and CC decreased from 5 to 1 mA. Results showed that the device containing the Si layer did not exhibit stability in the window; however, a distinguishable window greater than 10 was observed. Further, the typical RS role with the forming process, SET/RESET states, HRS/LRS during 10 switching cycles, cyclic stability of devices owing to W/CeO_2_ (20 nm)/Si (1 nm)/TiN as well as W/CeO_2_ (20 nm)/Si (2 nm)/TiN, respectively, were presented.

Moreover, the behavior of W/CeO_2_/Si/TiN is easily improvable by optimizing the thickness of the Si buffer layer. Furthermore, following the symmetric conducting channel model, we may describe the consecutive mechanism of rupturing channels depending upon the reset voltage. Hence, the abovementioned results may provide a suitable path for voltage control to optimize device performance and deep insight into the slow and steady reset process [87]. Figure 11c,d shows the SEM morphology with a graphic depiction of Ag/CeO_2_/La_0_._5_Sr_0_._5_CoO_3_ decorated over a sapphire substrate. It is obtained using the focused ion beam technique with a tungsten probe constantly mounted over the sample. Then, fabrication and investigation of Ag/CeO_2_/La_0_._5_Sr_0_._5_CoO_3_ comprising different levels are elaborated. The La_0_._5_Sr_0_._5_CoO_3_ (500-nm thick) and ceria (80-nm thick) layers were decorated over the sapphire substrate. Pulsed laser deposition at 700 °C was employed in an oxygen environment with a pressure of 150 mTorr. The pulse frequency in the laser ablation was set at 20 Hz and maintained with an energy density ranging from 2 to 3 J/cm^2^. A thermally evaporated 300-nm-thick Ag thin film was realized at normal temperature. The Ag/CeO_2_ micro interface was engineered through an ion beam over the La_0_._5_Sr_0_._5_CoO_3_ layer that exhibited renewability and reversibility in switching to an HRS state (OFF state) with insulating characteristics. By contrast, a semiconducting/metallic LRS state (ON state) showed resistance ratios up to 10^4^.

In this study, the formation of cells with micron and submicron values was more effective. The impact of micro-scaling associated with microdefects was evaluated at the cell boundary, and the electrical properties were evaluated through ion etching. The I–V curves of the Ag/CeO_2_/La_0_._5_Sr_0_._5_CoO_3_ cells with dimensions of 1–150 × 150, 2–15 × 30, 3–5 × 10, and 4–2 × 4 μm were measured. Ideal I–V results prior to the electrical forming process corresponding to different cell sizes are illustrated in Figure 11e,f. The Ag/CeO_2_/La_0_._5_Sr_0_._5_CoO_3_ structures were evaluated for breakdown voltage (which increased with a decrement in contact area). It is possibly caused by the existence of defects within the film. In this research, the high-resistance nature of the I–V results corresponding to Pool–Frenkel coordinates with Ln(*I*), V^1/2^ scaling was observed. In this process, the stimulating influence toward conductivity is explained by the Pool Frenkel effect, Schottky effect, and hopping conducting channels [88].

MnO/CeO_2_ heterostructures were prepared on Pt BE (150-nm thick) by RF magnetron sputtering at CeO_2_ and MnO targets in an Ar atmosphere at normal temperature. In addition, the MnO layer top area was decorated with an ~100-μm-diameter Ag TE by thermal evaporation with a shadow mask. Further, the Ag/MnO/CeO_2_/Pt cells were evaluated in terms of the stability in BRS characteristics, showing a high resistance ratio during the forming-free mechanism. In this way, the device always remains in HRS. Accordingly, a positive voltage applied on the TE will form the LRS, while a negative voltage on the Ag TE will lead to HRS, confirming and displaying the typical BRS behavior of these structures. In this case, the electrical forming process is not needed. Consequently, the forming-free process is associated with the creation of many interfacial states and defects into oxide layers [89].

Moreover, oxide materials such as NiO and CeO_2_ in the form of thin films are spin-coated over pure Si/ITO substrates. The reversible BRS showing optimal ON/OFF ratio has been reported to be ~10^3^. The bilayer species have exhibited a threefold improved ON/OFF ratio compared to the nanocomposite films. The charge carrier transport mechanism depends upon various p-n depletion structures. For composite films, the space charge field enlarges toward the inner p-n junction, leading to insensitive bias sweeping. In a study, the p-n junction was found to affect the production or rupture of CF channels [90]. The RS characteristics for memory devices depending upon the CeO_2-x_/La_0_._8_Sr_0_._2_MnO_3_ bilayer were also investigated. The BRS was evaluated in La_0_._8_Sr_0_._2_MnO_3_ at the micrometer scale and reproduced the results achieved at the nanoscale through conductive scanning force microscopy. Figure 11g presents the schematic illustration of the oxygen (O_2_^−^) ion migration mechanism in a metal/CeO_2-x_/La_0_._8_Sr_0_._2_MnO_3_ fabricated device. In the study, I–V characteristics corresponding to individual memristors were typically investigated under systematic voltage sweeps (0→ +V_max_ →0 → −V_max_→ 0). The measured curves of the CeO_2_/La_0_._8_Sr_0_._2_MnO_3_ structures were evaluated via a probing process over Ag electrodes as shown in Figure 11h. The forming process was required to induce BRS behavior and was established at low-voltage operation. Further, endurance efficiency with a resistance ratio of 10^2^–10^3^ at a reading voltage of −1 V was observed with consecutive memory functioning during 100 cycles as shown in Figure 11i. In addition, single La_0_._8_Sr_0_._2_MnO_3_ layers displayed stability in RS realized by metallic connections of probing operation in the form of oxygen exchange within the medium. Further, it was observed that inserting a CeO_2-x_ layer between two electrodes and the metallic-nature La_0_._8_Sr_0_._2_MnO_3_-layer prominently altered RS properties, thereby allowing the production of good microelectronics devices. In the as-prepared devices, the BRS trend was analyzed in a single La_0_._8_Sr_0_._2_MnO_3_ layer, where the SET/RESET processes were activated at minimal voltages with an enhanced resistance ratio. Therefore, the CeO_2-x_ layer (as an oxygen conductor) behaved like an O^2−^ reservoir, facilitating easy ion exchange with low-voltage operation [91].

The oxygen annealing effect attributed to the RS properties of bilayer TiN/ZnO/CeO_2-x_/Pt structures was analyzed. Schematic illustrations of the RS mechanism corresponding to un-annealing and annealing of TiN/ZnO/CeO_2-x_/Pt structures are presented in Figure 11j The switching trend for annealing and un-annealing effects on these devices revealed that with a small increment in the forming voltage, there may be a SET/RESET voltage drop. In this study, the endurance performance was critically improved in annealed oxygen ambient at an optimal temperature of 400 °C (with reduced O_V_s) in the ZnO/CeO_2-x_ film. Additionally, Schottky emission may have higher voltage limitations for annealed and unannealed (at 400 °C) structures. It is inferred that annealed devices in an oxygen environment improve the crystalline arrangement as the maximum number of defects are removed to improve the uniformity of RS characteristics at an appropriate forming voltage. Finally, the aforementioned upgrading relates to (Ce^+4^ to Ce^+3^) transition states. Accordingly, the transition may lead to O_V_s in the lattice creating charge balancing with an increase in their density in a bulk-type device interface. Hence, fewer CFs are produced, which is attributed to an allowed arrangement of crystallinity at the optimum annealing. Moreover, newly created CFs may provide an easy way for rupturing in the RESET process, thereby improving the memory window, as it is the basic need for improved nonvolatile memory characteristics [92]. Another bilayer TiN/HfO_2_/CeO_x_/TiN memristor has been reported to adopt RF magnetron-sputtering. The as-prepared devices were thermally annealed at various temperatures for performance advancement. Moreover, compared with unannealed devices, the effect on the coefficient of variation corresponding to SET/RESET voltages dropped by 35.1% and 59.4%, respectively. By contrast, the coefficients of variations in resistances attributed to resistances in LRS and HRS may decrease by 70.2% and 52.7%, respectively, under annealing at 400 °C for 2 min in an air medium. We performed I–V measurements depending upon the annealing effect by employing X-ray diffraction (XRD) and XPS analysis. It is concluded that the overall influence owing to grain boundary reduction and decreased O_V_ density in the switching layer is responsible for improved switching parameters of TiN/HfO_2_/CeO_x_/TiN devices. In this way, this study provides us with a simple way to enhance memristor performance [93].

## 7. Doped CeO_2_-Based Memristors

**Figure 12:**
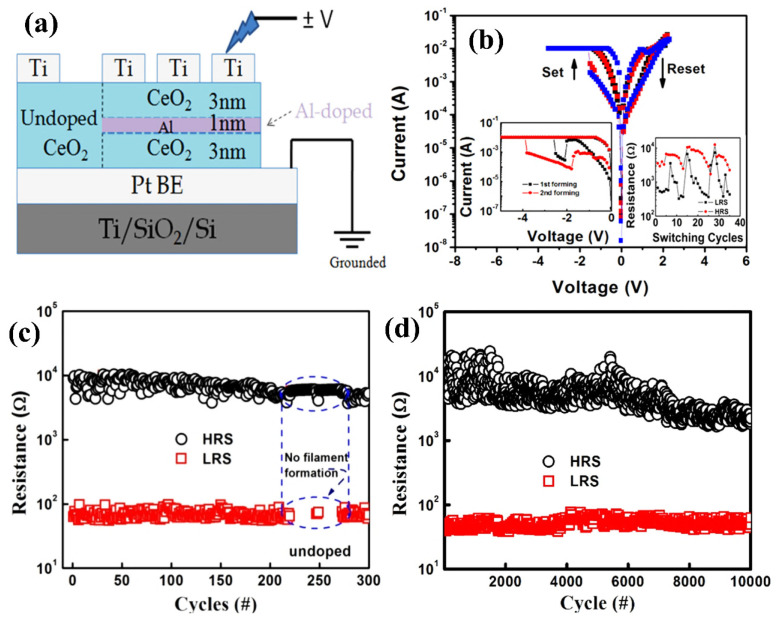
(**a**) Schematic illustration of Al-doped CeO_2_ structures, (**b**) Al-doped device behavior in negative-forming type with left- inset showing negative forming and right inset depicting the scattering in endurance. I–V characterization with endurance performances: (**c**) undoped and (**d**) doped devices. Adopted from [94] Copyright 2016 American Chemical Society.

The RS improvement trends in CeO_2_-based memristors have been analyzed through charge dynamics by using Al as the dopant. Here, results have indicated that Ti/CeO_2_: Al/Pt device sandwiching, shown in Figure 12a, always offers promising and attractive switching characteristics. In Figure 12b, an Al-doped device in the negative-forming mode is depicted by the left inset, while the right inset shows the variation in endurance. Further, the minimum forming voltage modulated the stability in SET/RESET voltages. In addition, an improved endurance (>10^4^) of switching cycles having window of R_OFF_/R_ON_ > 10^2^ was observed compared to the undoped Ti/CeO_x_/Pt structure, as shown in Figure 12c,d. The observed precision in the memory switching trend corresponds to a substantial decrease in the formation energy for O_V_s for increasing O_V_ creation in CeO_2_ layers corresponding to charge transfer as well as oxygen-obtaining capability of the Al dopant [94]. In this study, the Y^3+^-doped CeO_2_ memristor was analyzed for RS characteristics with operating current and voltage. This RRAM device was fabricated by forming Ce_1-x_Y_x_O_2-y_ ultrathin films using a photochemical metal–organic deposition strategy. It could be obtained with the direct patterning of Ce_1-x_Y_x_O_2-y_ films on a specific substrate via ultraviolet (UV) irradiation without photoresist and etching processes [95,96].

The photochemical metal–organic deposition procedure is utilized as it is comparatively stable against humidity and temperature owing to the beneficial photochemical metallic–organic precursor (main material), whereas chemical process is primarily initiated under UV irradiation. XRD as well as Raman spectrum results have increasingly revealed the occurrence of lattice shrinkage which induces and increases the structural alteration caused by O_V_ formation with the Y dopant. Ce_1-x_Y_x_O_2-y_ devices have exhibited bipolar filamentary behavior of RS and consequently both resistance states due to the decline in forming voltage with an increment in Y doping-induced rise in O_V_ density. However, the ON/OFF ratio decreases with an increment in Y density owing to reducing HRSs. Moreover, when the recovery of injecting oxide area associated with HRS switching is rendered, it causes defect sites comprising bounded electrons generated by yttrium. Spectro-microscopy revealed that ceria loses 24% oxygen during LRS, displaying a resistance variation of five orders of magnitude [97]. Three elements (Ca, Zr, and Gd) have been utilized as dopants in CeO_2_ film sandwiching into Pt/CeO_2_/Pt devices. In this case, the switching characteristic has been found to be unipolar. The electrical measurement examination for Gd and Zr dopants in the Pt/CeO_2_/Pt structure exhibited a resistance ratio of 104, improved retention stability with 104 s at 25 °C, and endurance of 1500 sweeping cycles.

By contrast, a Ca-doped device demonstrated the opposite trends. However, all results showed that chemical defects with O_V_ concentration in metallic Gd and Zr have a decisive impact compared to Ca doping in CeO_2_ films. Further, XPS analysis indicated vital oxygen-containing film activation in forming conducting channels, leading to resistance alteration within the conducting layer [98]. Further, the chemical-bonding energy obtained by XPS analysis revealed comparable undoped components; the Gd/Zr-doped cells possessed lower CeO_2_-bonding energy, leading to stability in configuration conversion. On the contrary, the Ca-doped components showed higher CeO_2_-bonding energy, leading to unstable configuration conversion. In conclusion, the said dopants in ultrathin films afforded resistance stability, and TEM results indicated that Zr dopant was much better than Gd dopant for the RS behavior of the CeO_2_-based RRAM devices. Thus, transition metals as dopants in CeO_2_ nonvolatile memory have shown improved memory functioning, better HRS/LRS ratio, attractive endurance, and fine retention capabilities. Therefore, with the overview of the experimental results, we can confirm that this memory is a promising candidate for future nonvolatile RS memory devices [99].

## 8. Neuromorphic Applications

The term “neuromorphic” was coined by Carver Mead to describe analog circuits that emulate biological neurons. Over recent years, the neuromorphic computing field has experienced remarkable advancement, including innovations in neuromorphic chip engineering that aim to replicate specific behaviors exhibited by components of the brain circuitry. Within the realm of neuromorphic circuitry, memristors have gained prominence owing to their resemblance to certain types of neurons [100].

### 8.1. Single-Layer and Bilayer CeO_2_-Based Synaptic Devices

Metal oxide-based memristors possess the potential for the advancement of scalable and highly efficient brain-inspired computing systems. Among these systems, oxide-based memristors display inherent electronic analog switching similar to biological synapses. For example, CeO_2_-based bilayer memristors occupying forming-free, low-voltage potential, and high energy efficiency and reliability play a vital role in biological synapses. Because of pulse measurement, analog behavior may be directly programmed in intermediate resistance states in such devices. Therefore, STDP may be implemented, strictly following spike-dependent Hebbian artificial learning. In this case, the memristive device exhibits substantial variation in the normalized synaptic strength (>30 times) while overlapping the presynaptic and postsynaptic neuron spiking. Accordingly, recent research has demonstrated a decisive role in the physical network with high density and excellent connection in neural networks [101].

### 8.2. Spike-Timing-Based Learning System

**Figure 13:**
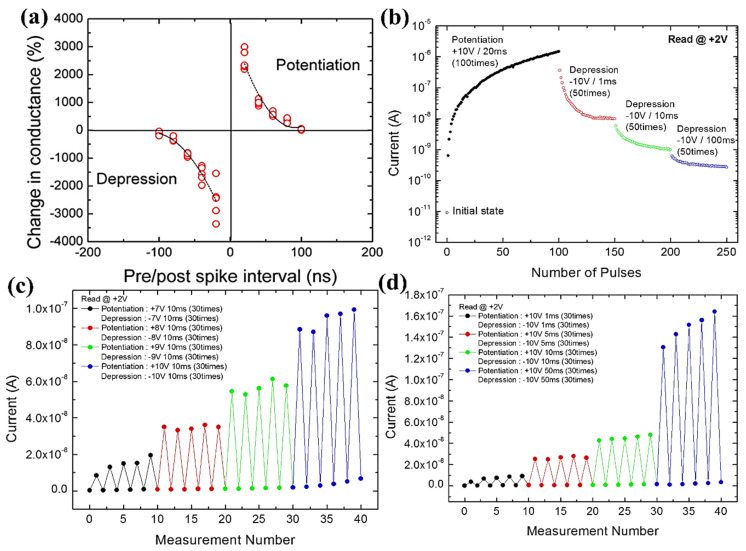
(**a**) Application of STDP learning process by utilizing HfO_x_/CeO_x_ memristors. Adopted from [101]. Copyright 2016 American Institute of Physics. Read current variation with repetition: (**b**) +10-V pulses of width 20 ms during 100 repetitions, and consecutive −10-V pulses of width 1, 10, and 100 ms during 50 repetitions, (**c**) pulses with increment in ±V (with fixed width), and (**d**) fixed ±10-V pulses (with varied width). Adopted from [18]. Copyright 2017 IOP Publishing.

Moreover, CeO_2_ with an HfO_x_ capping layer exhibits spike-based-learning operations that have shown novel behavior in terms of high efficiency and compactness for managing unstructured information. In these systems, the learning process is necessarily followed by spike-based Hebbian learning, proving that it is STDP, as it clearly describes the synapse strength variation during a time interval corresponding to presynaptic and postsynaptic neuron spiking. To perform these activities, a mean spiking rate of 1 MHz (10^5^ times) is evaluated in the human brain. Moreover, the period is considered as 1 s to refresh the neurons’ existing position while calculating synaptic currents, whereas neuron-spiking probability is taken as 0.01 in the central nervous system. In Figure 13a, the plot of normalized conductance variation versus time difference of the pre/post-spike interval demonstrates synaptic behavior. The fitted data allied with exponentially decaying functions evidences the STDP behavior analogous to the human brain synapse. Further, the data showed a significant variation corresponding to normalizing conductance in this device, which was greater than 30 times in the state of overlapped pre/postsynaptic spikes [101].

### 8.3. Potentiation and Depression Behaviors

A symmetric electrode, such as a Pt/CeO_2_/Pt device, has demonstrated synaptic-dependent potentiation and depression behaviors showing polarity-oriented analog switching. Here, the synaptic weight variation is controlled by parameters such as amplitude, width, and pulses, resulting in an artificial synaptic device for neuromorphic computing systems [18]. Figure 13b illustrates the steady and successive current increment at a read voltage of +2 V with repeated pulses of amplitude +10 V and a pulse width of 20 ms with 100 repetitions. This study revealed the analog transition with an HRS-to-LRS ratio > 10^5^, which is better than already reported data. Because of reversible switching, LRS returns to HRS with the application of repeatable −10 V pulses with widths of 1, 10, and 100 ms during 50 repetitions. By contrast, the current with a read voltage of +2 V under low voltage pulsing was 10^−10^ A, and read disturbance became negligible. The analog decrement and increment in resistance followed the potentiation and depression in synaptic tendency, respectively. More importantly, the optimization in resistance variation, which is ~1 MΩ–200 GΩ), permitted the synapse toward extensive limitation in synaptic weight functioning to generate a signal, thereby establishing learning and memory functions.

The current variation may be controlled by the voltage pulse amplitude along with the width, as illustrated in Figure 13c,d. In addition, the current is generated with a read voltage of +2 V during higher voltage pulsing 30 times. At a specific pulse width of 10 ms, the current dynamics at LRS may increase (~20 to 100 nA) with the pulse amplitude increment of +7–+10 V, as shown in Figure 13c. Likewise, the current may increase by ~10–170 nA when the pulse width is increased from 1 to 50 ms under a specific pulse amplitude of +10 V, as demonstrated in Figure 13d. Moreover, both cases maintain the reversible HRS at negative voltage pulses. The retention mechanism for guessing short-term/long-term synaptic memory with the pulsing operation. With the current increment (tens of times) with positive potentiation pulsing, there may be a decrement in retention with time, leading to memory loss. Interestingly, it decreased speedily in the initial 30 s, whereas it decreased more slowly thereafter. However, the current may be four or five times greater than the primary measurement after 10 min. The result may be more significant/comparable when retention becomes favorable for short-term memory in biotic systems analogous to the human brain, caused by momentary recalling of information during the processing time that usually lasts (seconds to tens of minutes) [102]. On the contrary, improved retention may be mandatory during the long-term memory (LTM) process. In addition, the memory loss in the system may be considered to be explored through irregular electrical charging/recovery (Ce^3+^ to Ce^4+^), thereby resulting in the annihilation of O_V_s when O^2−^ are recombined. The O^2−^ ions must instantaneously stop moving toward the Pt electrode to prevent this recombination. Consequently, the memory loss mechanism and improved retention properties are still questionable [18].

### 8.4. Potentiation Motion and Synaptic Weight Decay

**Figure 14:**
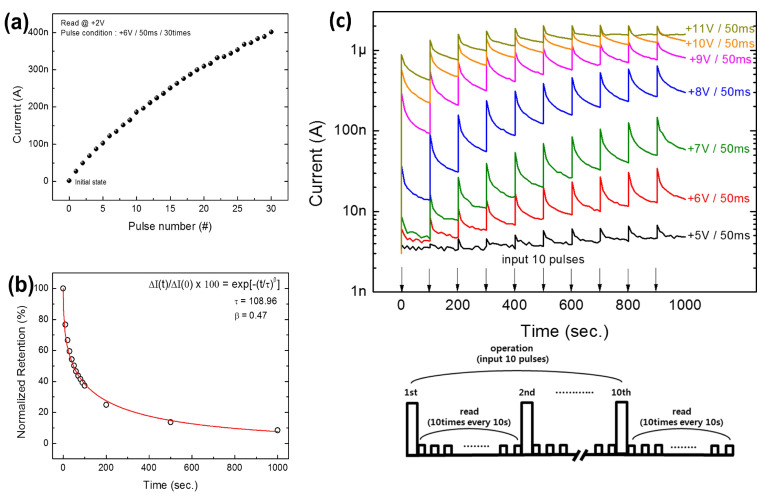
(**a**) Read current at +2 V) with pulsing at +6 V, width of 50 ms, and time interval of 2 s for 30 cycles; (**b**) the subsequent normalized memory retention ((ΔI(t)/ΔI(0)) × 100(%)) along with time fitting with stretching relaxation function exponentially; and (**c**) the read current at +2 V with time in the form of repeated application of voltage pulses with different amplitudes (+5–+11 V), 50-ms width, and 100-s time interval. Adopted from [20]. Copyright 2018 IOP Publishing.

Figure 14a illustrates the consecutive increment in current at +2 and +6 V during repeated pulses and width of 50 ms with 30 repetitions in potentiation dynamics, and Figure 14b shows normalized memory retention when potentiation is slowly reduced during time *t*. Therefore, synaptic weight decay is equivalent to the human memory–forgetting curve, the STP, whereas the gradual decay of final synaptic weight denotes LTP [102]. On the contrary, biotic synapses manage the input information and correspondingly rearrange the memory states toward a synapse. Figure 14c illustrates a multistore model system for human psychological memory, as presented in the work of Atkinson and Shiffrin. Furthermore, in this model, the external information may be assessed and later stored for various hierarchy stages, thereby originating from sensual memory, momentary short-term memory (STM), and perpetual LTM by attention learning associated with repetition/rehearsal progressions. In addition, Figure 14 illustrates the enlarged current with memory transition under repeating input pulses to verify the rehearsal scheme, and the trend of STM toward LTM transition with repeated pulsing processes coincides with the multistore model as shown in Figure 14c. Moreover, the STM-to-LTM transition has been constantly detected by increasing the pulse width.

Further, quantitative analysis has revealed the distinguished rehearsal effect to enhance memory retention. The gradual decrement in retention with time and the decaying rate significantly decreases with increasing number of stimulation (N). Herein, the rehearsal mechanism with repeating pulses (N = 70) may significantly enhance the synaptic weight variation as 75–618 nA, while the relaxation time is 24–580 s, and is firmly following the tendency reported by Chang et al. [102], as the resistance variation due to O_V_ diffusion is improved and stabilized under repeated stimulation. In addition, the memory retention curves are closely fitted, which is attributable to the stretching relaxation function exponentially depending upon the relaxation time. Thus, depression is confirmed by the pulsing mode results from a quicker and unexpected decaying current closer to the original state, while the retention loss with time results from more gradual incompleteness in memory loss. Hence, synaptic weight modulation in connection with stable memory is caused by memory loss with time, which may be strengthened by increasing the pulse frequency.

### 8.5. Short- and Long-Term Plasticity

Analog synaptic weight modulation has been proved to show linearity and symmetric behavior. Therefore, it demonstrates LTS through resistance variation in a Pt/ITO/CeO_2_/Pt memristor rather than Pt/CeO_2_/Pt memristive memory, possessing nonlinearity and asymmetric resistance variations. The same resistive memristor displays linearity and symmetric resistance variation trends associated with various voltages with opposite polarities toward synaptic potentiation and depression functions. Additionally, the memristor also exhibits considerable LTS concerning controllable synapse weight with time, which may be extracted from the capping layer (ITO). It behaves like a source of OIs in the CeO_2_ layer for retaining resistance variation. In the previous literature, biological synapses have exhibited memory-forgetting time depending on input levels. Here, a comparative study of memristors without and with a capping layer may elucidate the proper functioning of the ITO layer in terms of linearity, symmetric nature, and long-term stable behavior in resistance variation in these memristors for application in artificial synapses in neuromorphic computing [19].

By contrast, classic nonvolatile memory may disclose long-term retention of memory levels. Additionally, the current decline over time in the potentiation process for the Pt/CeO_2_/Pt device and Pt/ITO/CeO_2_/Pt memristor is considered comparable. The current decay comparison with identical ranges and pulses of various amplitudes is performed in these memristors. Primarily, the reference memristor demonstrates voltage pulses as +6–+9 V with a corresponding width of 50 ms applied for 10 cycles, and subsequently, the read current generated by +2 V during 10 cycles in 10 s. Further, to analyze the current decay during 100 s, all steps are repeated again 10 times. The above procedure repeats itself in memristor functioning by utilizing a pulse amplitude of +8–+11 V. Further, with the current increment associated with potentiation pulses, both memristors display current-decay functioning during specific intervals, representing forgetting operations based on biotic memory. Moreover, the quick current decay, analogous to STP, shows a gradual decline toward LTP [102]. After decay for 100 s, further increment in the current is observed with the following pulse, corresponding to PPF features, which are characterized by additional synaptic weight by applying two close consecutive input pulses. Application of a high pulse amplitude will result in less current decay associated with enhanced PPF. Furthermore, the retention characteristics are then investigated by measuring current in the absence of potentiation pulses.

**Figure 15:**
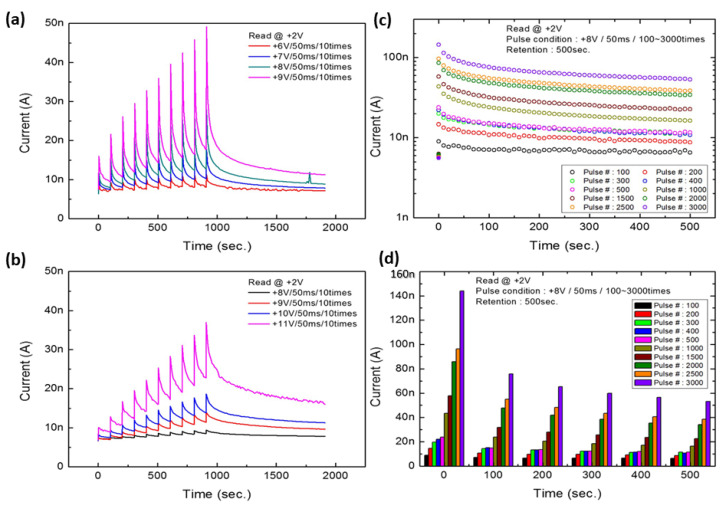
Schematic display of read current dependence on the decaying function during subsequent potentiation, with PPF, and retention stability in 1000 s for (**a**) Pt/CeO_2_/Pt reference device and (**b**) bilayer Pt/ITO/CeO_2_/Pt cell. Schematic of read current at +2 V potential for to Pt/ITO/CeO_2_/Pt on subsequent application of +8 V pulses, along with growing pulse repetitions from 100 to 3000. (**c**) Semilogarithmic scale and (**d**) histogram at specific times with a 100-s interval. Adopted from [19]. Copyright 2019 American Institute of Physics.

In Figure 15a, the reference Pt/CeO_2_/Pt memristor displays current increment values as 7.5–49 nA under +9 V pulses and later decreases to 11 nA with a retention time of 1000 s. By contrast, the Pt/ITO/CeO_2_/Pt memristor achieved high in retention performance. In particular, the current increases from 6.9 to 37 nA during the potentiation process, which subsequently declines to 16 nA during 1000 s, as depicted in Figure 15b. Surprisingly, retention the results of both memristors are prominently connected with the resistance variation mechanism, which is thus difficult to reveal owing to instantaneous resistance change by applying a voltage that in turn decays over time. In this respect, retention operation in favor of the double-layer Pt/ITO/CeO_2_/Pt device is enhanced by increasing the pulse repetition number n, as demonstrated in Figure 15c,d. For instance, the current-increase trend shows an ΔI(0) of ~140 nA generated with 3000 repetitions, followed by a decaying trend, with ΔI(t) = ~50 nA during 500 s. On the contrary, ΔI(0) becomes ~3.3 nA during 100 repetitions with the pulse rate, and ΔI(t) = ~0.8 nA during 500 s. Finally, speedy decay with a smaller n value is attributed to STM features.

Moreover, better retention with increased n values consistent with LTM, significantly mimicking identities during the transition from STM to LTM, is realized in several memory hierarchy stages in the natural brain. Figure 15c demonstrates the linear scaling for the repeating pulses caused by a larger ΔI, resulting in speedy decay at the preliminary level despite the retained window becoming larger. Subsequently, it contracts the gap among the current stages corresponding to repeating pulse number. Despite the decaying process, the current stages are distinct, confirming the multilevel probability or analog synaptic weight modulation concerning repeating pulse numbers. Thus, analog characteristics such as linear behavior, symmetric structure, and prolonged stability in synaptic weight modulation are successfully investigated for the memristive device under discussion. In addition, more parameters such as operating speed, voltage level, and power consumption are considered critical factors in the synaptic procedure. The appropriate engineering of the aforementioned factors may be critically performed in applications for artificial synapses and neuromorphic computing [19]. Artificial synapses with analog resistance variations are actively investigated because they are fundamental elements for brain-inspired neuromorphic computing.

Recent information has suggested interface-state-oriented artificial synapse modulation. In particular, analog behavior, linear trend, and symmetric synaptic weight in Pt/CeO_2_/Pt devices are based on subsequent deposition on a heated CeO_2_ layer. Further, memristive characteristics have been studied at optimized (400 °C and 500 °C) annealing temperatures, showing consistent and analog-nature resistance decrement at positive polarity and reverse at negative polarity. This critical resistance alteration is extensively responsible for linearity and symmetry during potentiation and depression functioning, which may be tunable through variation in pulse number with amplitude. In addition, STP and LTP have also been investigated under different pulse conditions. In addition, conducting channels are tailored through the Schottky conducting phenomena caused by energy barrier junctions. Moreover, minor resistance variations have been investigated in the reference memristor, but with the controlled insertion of SiO_2_ interfacial layers at both electrodes concerning the Pt-CeO_2_ junctions, thereby fabricating Pt/SiO_2_/CeO_2_/SiO_2_/Pt structure. Here, it has been concluded that the resistance variations are strictly voltage-driven, thereby tuning the Pt/CeO_2_ junctions. Necessarily, controllable interfacial layers are considered to be decisive toward synergetic synaptic operations [26].

### 8.6. Pavlov’s Dog Experiment-Based Synoptic Study

More recently, an artificial neural network has been considered the hottest research spot for advanced science and technology. The CeO_2_ exhibits excellent performance among various oxide-based materials, such as longer retention with healthier stability. However, its use in artificial neural synapses is still questionable. In this regard, a Ag/CeO_2_/Pt memristive-type device has been realized toward the selected target. Herein, the artificial synaptic functions are successfully explored, and a sequential neuromorphic system simulation has been updated. In addition, the relations among the pulse arrangement parameters involving resistance states owing to synapse devices have also been investigated.

A study tailored an electrically generated signal simulation during Pavlov’s dog experiments. These findings demonstrate the device’s significance for realizing important applications in artificial neural synaptic simulations [39]. To achieve these targets, bionic synaptic appearances are analyzed afterward. The biological synapses show that excitatory neurotransmitters are released through activation of the presynaptic sheath, which produces current reverting into the postsynaptic sheath. Here, Ag acts as the neurotransmitter to connect both electrodes (top and bottom) under application of a biased voltage signal. Then, the current is abruptly increased by using the voltage signal at the TE. When the write voltage of 0.275 V is removed, the current returns to the starting level prior to stimulation during a fixed time, thereby exhibiting STP trend, as the excitatory postsynaptic current is produced at that level.

**Figure 16:**
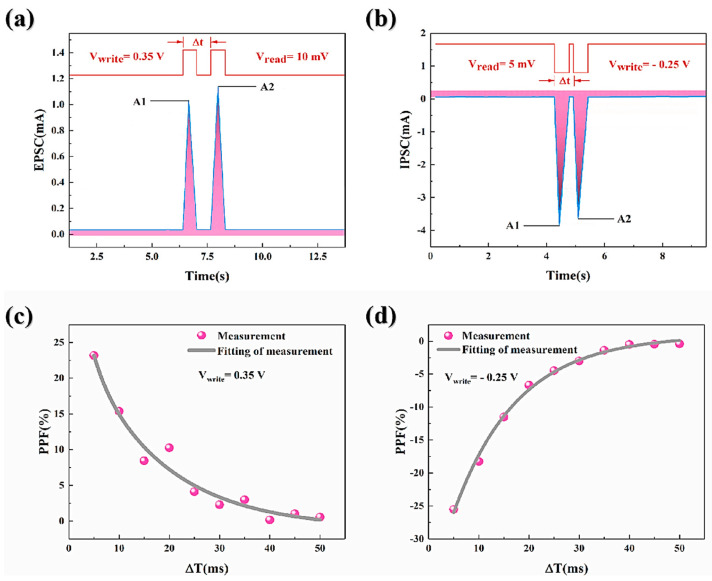
Pair of pulse waves with (**a**) positive signal simulation and (**b**) negative signal simulation. Fitting consecutive outcomes with (**c**) positive and (**d**) negative pulses. Adopted from [39]. Copyright 2022 Elsevier B.V.

To demonstrate the further relationship between time and the attenuation owing to excitatory postsynaptic current, Figure 16a,b shows that two consecutive pulse signals are intended to simulate PPF, significantly exploring STP movement in the neuroscience literature. Moreover, to simulate potentiation, two consecutive positive pulses are operated; thereby, 0.35 V is achieved in the writing process. Furthermore, to simulate the depression effect, two negative pulses must be applied while achieving a V-write of −0.25 V. Moreover, both simulated synaptic activations indicate that the current is read out through a small voltage amplitude, which is called V-read. The aforementioned synaptic trend usually occurs when double-stimulated signals are generated during a short time. These studied synaptic weights are given by Equation (1). The curve is fitted using Equation (2). Here, pulse stimulations are illustrated in Figure 16c,d, where weight updates are.

Δ*W* = (*G*_2_ − *G*_1_)*/G*_1_, (1)

PPF = (*G*_2_ − *G*_1_)*/G*_1_ × 100% = *C*_1_*exp*(−*t*/*τ*_1_) + *C*_2_*exp*(−*t*/*τ*_2_). (2)

*G*_1_ and *G*_2_ denote the first and second pulse conductance obtained from the current measurements (A1, A2), as shown in Figure 16a,b. Moreover, *t* denotes the pulse interval, and *C*_1_ and *C*_2_ indicate the first facilitation values for the concerning phases; *τ*_1_, *τ*_2_ represent conductance magnitudes for the first and second pulses as a representative of synaptic weights. At the forward signal, *τ*_1_ and *τ*_2_ values are observed as 2.74 and 15.94 ms, respectively. At a negative signal, the achieved values of *τ*_1_ and *τ*_2_ are 12.7 and 12.71 ms, respectively. The logic behind the large gap between *τ*_1_ and *τ*_2_ (under positive pulses) is the easier formation of CFs than fractures, resulting in fast activation in the synaptic study. To increase the response current, the pulse interval is reduced so that successful simulation of the impact of consecutive signals and signal duration law in neurosynaptic trends may be achieved. As in the previous literature, synaptic plasticity may be characterized as STP and LTP. STP may merely be continued for a short time after pulse signals. On the contrary, LTP may extend to several hours/days without resistance variation. Figure 16c,d demonstrates device conversion (STP to LTP) when a pulse cluster with 0.1-V amplitude, 20-ms interval, with 5-ms duration is applied. The response current abruptly enhances when a pulse is applied and quickly returns to the previous state. Again, when a pulse group with 0.3-V amplitude and 5-ms interval and 20-ms duration is applied, there may be a gradual decrease in the response current. Upon removing the pulse, the response current still shows stability, whereas the resistance shows a lower value than the initial level; conversion from STP to LTP is also accomplished.

**Figure 17:**
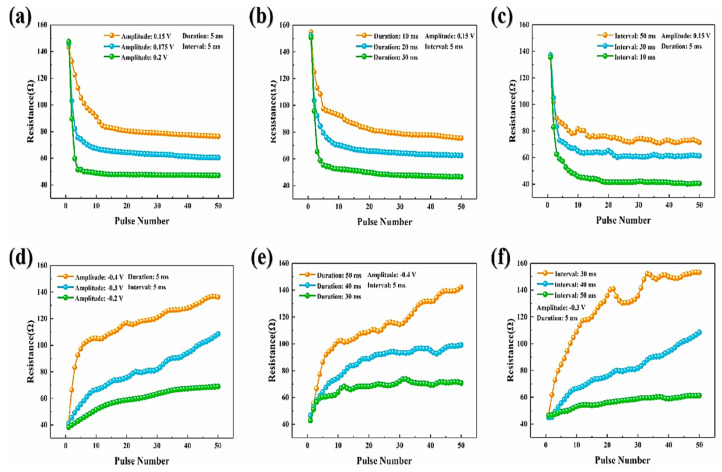
Relationship among three pulse train variables with variation in the resistance state with (**a**) positive pulse train amplitude, (**b**) time interval, (**c**) duration, (**d**) negative pulse train amplitude, (**e**) time interval, and (**f**) with duration. Adopted from [39]. Copyright 2022 Elsevier B.V.

Further, the associations among pulse train variables, such as duration, amplitude, and interval, correspond to resistance states. The device resistance is examined by using these controllable variables, as depicted in Figure 17a–c, showing their effect on resistance modulation for forward stimulation; further, Figure 17d–f demonstrates the effects under negative stimulation. The impact of speed and degree of resistance variation is positively linked with the with pulse amplitude in specific intervals, whereas they are negatively attached in pulse interval trains. These results mimic learning by human intelligence. For instance, when an individual tries to recall some memory, the same individual may easily forget if the recitation intensity is inadequate.

**Figure 18:**
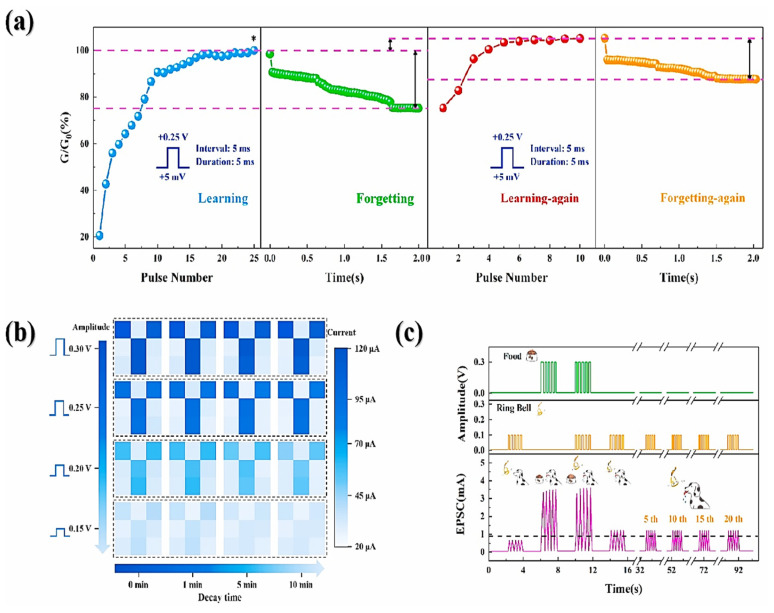
(**a**) Schematic of the simulation of multiple learning along with forgetting responses in the natural brain, whereas * indicates maximum learning behavior to be achieved (**b**) patterning the memory of the synaptic device array corresponding to the memristor’s derivatives, (**c**) Pavlov’s dog experimental simulation by training the device with consecutive pulse signals. Adopted from [39]. Copyright 2022 Elsevier B.V.

On the contrary, LTM may be comprehended when the recitation intensity may strengthen. Consequently, the resistance level may vary intensely upon the application of a forward pulse. Interestingly, only a few pulses are required to implement the transition of the resistance state, which is caused by Ag atoms not returning to the TE during the reversing reset voltage. Thus, STP, LTP, training learning, and complex learning with forgetting functioning in the biotic brain may be simulated. In this regard, forgetting roles analogous to those in the natural brain are used to understand the forgetting process. First, a learning pulse is applied 25 times, whereby the optimum conductance after the initial learning process is proposed to be 100% for the memory intensity and is analogous to human learning behavior, identical to first-time learning as shown in Figure 18a. Then the conductance is read every 5 ms after removing the learning pulse. It is significantly achieved by memory intensity detection, which decays quickly at first, indicating a similar trend as that in human STM. Nevertheless, memory intensity will stabilize gradually during increasing time intervals, finally maintaining 75% retention intensity, attributed to human LTM. Afterward, the identical pulse learning signal is repeated.

When the learning pulse is removed in an identical time interval, the device’s memory intensity attenuation becomes lower than the initial level. By contrast, stabilized learning intensity becomes higher than the initial level. This is analogous to the human brain, as it could attain stronger memory than in early times by repeating learning processes caused by forgetting during specific periods. Figure 18b demonstrates retention array information, which could be achieved by increasing the pulse amplitude, whereas writing data intensity may be stronger than the previous level. This way, the device is realized to find short-term and long-term neuro status toward storage and memory fields.

In addition, bionic learning (Figure 18a) of the device is impossible by employing pulse training experiments intended to simulate familiarity testing (Pavlov’s dog). Figure 18b shows that the pulses group with 0.1-V amplitude is utilized for simulating ringing stimulation, while the pulses group with 0.3-V amplitude is utilized for simulating food stimulation; the excitatory postsynaptic current is used for the simulation of the dog’s response stimulation. Here, a current of 0.9 mA is set as a reaction boundary; the response current may be produced through a stimulation greater than 0.9 mA. Then, the reaction of the device is considered to be drooling for the dog learning.

Further, upon application of the bell-pulse stimulation, the memory does not show an optimum responsive state. However, the device exhibits a higher response state upon using food pulse stimulation. Then, ring pulse as well as food pulse are executed to simulate dog training course. This time, the device exhibits a higher responsive state. Furthermore, in training, when a bell-pulse stimulus is used, the device reaches a higher responsive state and maintains the running state during subsequent 20 bells stimulation, as shown in Figure 18c. Consequently, the desired memory positively simulates the standard Pavlov’s dog experiment comprising highly learned efficiency with excellent memory features [39].

### 8.7. Latest Development in Ceria-Based Neuromorphic Computing

**Figure 19:**
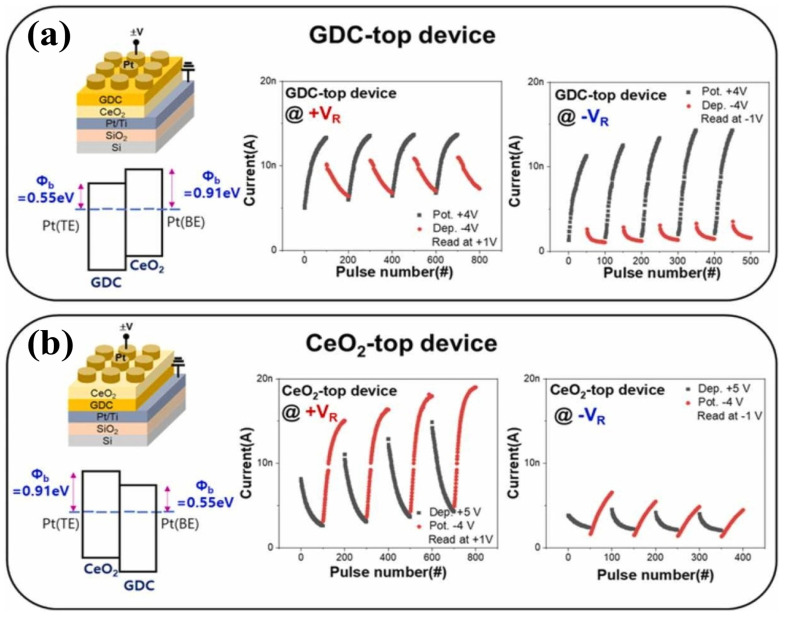
Proposed bilayer memristors containing a stacking structure of CeO_2_ and GDC layers and their linear, symmetric, and analog synaptic weight update characteristics depending upon their stacking order. The synaptic characteristics/functions in the form of potentiation and depression outcomes for the (**a**) GDC-top device and (**b**) CeO_2_-top device for consecutive potentiation/depression functions [103]. Copyright 2023 Elsevier B.V.

In this section, we explore potentiation and depression behaviors that exhibit remarkable linearity and symmetry. These characteristics arise owing to the incorporation of a lower concentration of O_V_s within the Gd-doped ceria (GDC) layer when combined with CeO_2_. This leads to oxygen vacancy redistribution and unique synaptic weight redistribution behaviors. The distinct trends in synaptic weight update for polarity-dependent potentiation and depression voltages are attributed to the stacking order of CeO_2_ and GDC as depicted in Figure 19. We demonstrate how potentiation and depression functions can be studied using +4V−4V as potentiation/depression voltages for the GDC-top device and +5V−4V for the CeO_2_-top device, each with distinct consecutive functions at corresponding voltages. These behaviors are further explained through voltage-controlled energy barrier modulation at the junction of CeO_2_ and GDC or at the Pt electrode junction, which is influenced by oxygen vacancy redistribution.

Furthermore, we showcase a theoretical simulation that employs an improved handwritten digit patterning approach via a bilayer perceptron neural network. This simulation yields an average accuracy of 88%, encompassing dynamic range, linearity, symmetry, and accuracy of states. The synaptic properties explored are also demonstrated through 32 × 32 crossbar arrays of GDC-top memristors, underscoring the potential of two-layer memristors in pioneering the implementation of artificial synapse networks for novel applications in neuromorphic computing [103].

**Figure 20:**
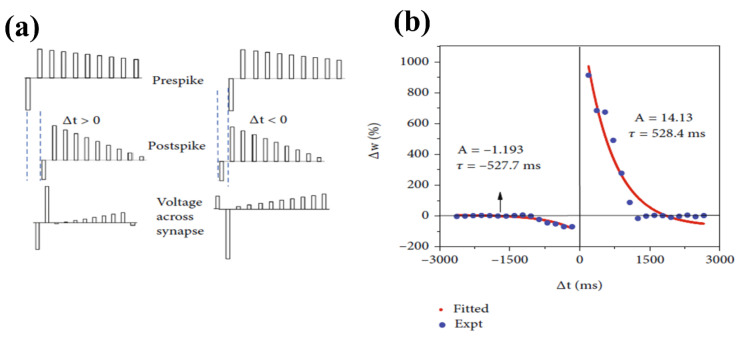
Demonstration of (**a**) the STDP realization schemes with pulse amplitude modulation. The pulse amplitudes for the pre-spike reveal the values −0.5, 0.45, 0.43, 0.41, 0.39, 0.37, 0.35, 0.33, 0.31, and 0.29 V. For the post-spike, the values are −0.25, 0.45, 0.4, 0.35, 0.3, 0.25, 0.2, 0.15, 0.1, and 0.05 V. The pulse width of the pulses is 50 ms. The pulse interval between consecutive pulses is 150 ms. (**b**) The application of STDP learning for the Pt/Ti/AlO_x_/CeO_x_/Pt memristor synapse [104].

For biological synapses, one of the most crucial neuromorphic characteristics is STDP. STDP demonstrates that when the pre-spike signal precedes the post-spike signal (Δt > 0), it leads to long-term potentiation (LTP), resulting in an increase in synaptic weight (w). Conversely, if the post-spike follows the pre-spike (Δt < 0), long-term depression (LTD) occurs, leading to a decrease in synaptic weight (w). Figure 20a depicts the waveform designed to induce the STDP phenomenon using continuous single pulses. Notably, the first time slot is occupied by a negative pulse, while a positive pulse with reduced amplitude appears in the subsequent time slot. When pre-spike and post-spike signals overlap, the programming pulse corresponds to the necessary amplitude for resistance modulation. Additionally, the voltage drop across the device arises from the disparity between pre-spike and post-spike voltages. When spike timings are closely aligned, the negative pulse often overlaps with the positive pulse, yielding a higher amplitude and consequently more significant resistance variation. If the pre-spike precedes the post-spike, a positively programmed pulse emerges (left side of Figure 20a), whereas the opposite results in a negatively programmed pulse (right side of Figure 20a).

Furthermore, Figure 20b showcases the simulation of the STDP learning rule applied to the current device. For Δt > 0 with Δw > 0, LTP is observed, whereas for Δt < 0 with Δw < 0, LTD is significantly induced. However, tighter spike timings result in more substantial resistance modulation. Notably, experimental learning data following the STDP rule reveals an impressive fit with exponential functions, supported by fundamental fitting parameters, as also demonstrated in Figure 6b. The equation capturing this relationship is provided as follows:

(3) $$\Delta W=Aexp\left(-\frac{\Delta t}{\tau }\right)+\Delta w0,\;$$

In this context, the symbol ΔW represents the updates in synaptic weight. A is employed as a scaling factor, and τ signifies the time constant. Moreover, the device’s adjustable conductance, synaptic saturation, and simulation of the STDP learning rules collectively demonstrate the considerable potential of Pt/Ti/AlO_x_/CeO_x_/Pt storage devices for adaptive features within neuromorphic systems. Consequently, the device exhibits synaptic characteristics, including adjustable conductance, synaptic saturation, and the ability to simulate STDP learning rules. These aspects highlight its significant potential for application in artificial neuromorphic computing [104]. Copyright © 2022 Wenqing Wang et al.

Following this comprehensive discussion on recent advancements in the field of neuromorphic computing based on ceria, we aim to delve into the domain of neuromorphic dynamics linked with NWNs. This area has also explored brain-inspired neuromorphic systems, wherein synapse-like interfaces establish neural networks to facilitate artificial learning processes, as elucidated by Nakayama et al. [105] Notably, NWNs epitomize an exemplary neuromorphic system. These networks can be categorized as metal/metal-oxide NWNs, atomic switch NWNs, and metal/polymer NWNs. Each of these systems comprises a network and exhibits switching behavior that hinges on the choice of materials or schemes employed [105].

In conclusion, it is imperative to acknowledge that the abovementioned work offers a level of flexibility, particularly in the context of ceria-based neuromorphic nanowires. This flexibility serves as an encouraging factor for researchers exploring this intriguing field.

## 9. Conclusions and Perspective

High-k oxide-based materials are extensively suggested as promising memristors; however, they are still challenging in neuromorphic studies. CeO_2_ has become more efficient and advanced in RRAM technology, as it covers the maximum gap left behind in the memory field. Therefore, CeO_2_-based memristors have been widely used because they show substantial behaviors in the filamentary mechanism and classification, optimum switching behaviors (unipolar, bipolar, and threshold) depending upon the nature of electrodes (symmetric and asymmetric), and switching layers (single with capping layer, and doped-insulating layer). In this context, various RS devices have been discussed in detail in this review. Further, because of their highly efficient behavior in the RS mechanism, CeO_2_-based synaptic devices have been suggested in this work, exhibiting unique neuromorphic characteristics compared to other high-k oxide-based memristors. These devices afford optimum RS behavior and improved outcomes in their synaptic behavior for neuromorphic artificial systems. Therefore, this review strictly emphasizes CeO_2_-based artificial synaptic devices. They are based on spike-based learning, potentiation, and depression, STP and LTP, potentiation motion, and weight decay. Finally, Pavlov’s dog experiments for the advanced synaptic study of CeO_2_-based memristors are systematically focused on, revealing recent advances in synaptic devices. High-k oxide materials may be the best alternative to compensate for the weak areas indicated in this review.

## Data Availability

Not applicable.
